# Ribonuclease inhibitor and angiogenin system regulates cell type–specific global translation

**DOI:** 10.1126/sciadv.adl0320

**Published:** 2024-05-31

**Authors:** Martina Stillinovic, Mayuresh Anant Sarangdhar, Nicola Andina, Aubry Tardivel, Frédéric Greub, Giuseppe Bombaci, Camille Ansermet, Marco Zatti, Dipanjali Saha, Jieyu Xiong, Takeru Sagae, Mariko Yokogawa, Masanori Osawa, Manfred Heller, Adrian Keogh, Irene Keller, Anne Angelillo-Scherrer, Ramanjaneyulu Allam

**Affiliations:** ^1^ Department of Hematology and Central Hematology Laboratory, Inselspital, Bern University Hospital, University of Bern, Bern, Switzerland.; ^2^ Department for BioMedical Research, University of Bern, Bern, Switzerland.; ^3^ Graduate School for Cellular and Biomedical Sciences, University of Bern, Bern, Switzerland.; ^4^ Graduate School of Pharmaceutical Sciences, Keio University, Minato-ku, Tokyo, Japan.; ^5^ Department of Visceral Surgery and Medicine, Inselspital, Bern University Hospital, University of Bern, Bern, Switzerland.

## Abstract

Translation of mRNAs is a fundamental process that occurs in all cell types of multicellular organisms. Conventionally, it has been considered a default step in gene expression, lacking specific regulation. However, recent studies have documented that certain mRNAs exhibit cell type–specific translation. Despite this, it remains unclear whether global translation is controlled in a cell type–specific manner. By using human cell lines and mouse models, we found that deletion of the ribosome-associated protein ribonuclease inhibitor 1 (RNH1) decreases global translation selectively in hematopoietic-origin cells but not in the non–hematopoietic-origin cells. RNH1-mediated cell type–specific translation is mechanistically linked to angiogenin-induced ribosomal biogenesis. Collectively, this study unravels the existence of cell type–specific global translation regulators and highlights the complex translation regulation in vertebrates.

## Teaser

Ribosome-associated protein ribonuclease inhibitor 1 (RNH1) specifies hematopoietic-specific translation in mammals.

## INTRODUCTION

Gene expression diversifies the phenotype and functions of cells in multicellular organisms. Primarily, gene expression is regulated at the transcriptional level through the action of transcription factors (*1*). While translation is crucial for decoding information from mRNAs to produce proteins, it has traditionally been seen as a default process without direct regulation of specific gene expression. However, recent studies indicate that translational control plays a crucial role in fine-tuning protein synthesis and modulating the expression of specific mRNAs (*2*). For example, a mutation in the ribosomal protein L38 (*RPL38*) gene in mice deregulates the translation of a subset of homeobox mRNAs, leading to tissue-specific patterning defects (*3*). Further, mutations in several ribosomal protein (RP) genes, such as *RPS19*, *RPL5*, *RPL11*, *RPL35A*, *RPS10*, *RPS17*, and *RPS24*, cause Diamond-Blackfan anemia (DBA) (*4*). DBA is characterized by bone marrow failure, anemia, and craniofacial malformations (*5*). Studies have shown that decreased translation of certain mRNAs involved in erythroid differentiation is responsible for the anemia phenotype in DBA (*6*, *7*). RPs are core components of ribosomes and are present in every cell. Mutations in their genes should cause global phenotype, but what accounts for these tissue-specific phenotypes after translational defects is not completely understood. Several mechanisms have been proposed to address this and are matter of debate (*8*–*11*). Although specific mRNA translation adds another layer of gene expression regulation, it is not yet known whether global translation is controlled in a cell type–specific manner.

Ribonuclease inhibitor 1 (RI or RNH1), also known as ribonuclease (RNase)/angiogenin (ANG) inhibitor, is a ubiquitously expressed protein in mammalian cells (*12*, *13*). Phylogenetically, *RNH1* has no apparent homologs in invertebrates and lower vertebrates (fig. S1), suggesting that it is a higher vertebrate-specific gene. It evolved via exon duplication and is conserved among mammals (*14*). RNH1 is known to bind and inhibits pancreatic-type RNases such as RNase 1, RNase 2, RNase 4, and ANG (also known as RNase 5) (*12*). RNH1 is composed entirely of leucine-rich repeats (LRRs) and displays vast surface areas that foster protein-protein and protein-ligand interactions (*12*). Consistent with this, RNH1 has been reported to have other biological functions besides its RNase inhibitor role, such as binding and inhibiting the endoplasmic reticulum stress sensor IRE1 activity (*15*), involvement in cancer growth and metastasis (*16*), tRNA degradation (*17*), microRNA-21 (miR-21) processing (*18*), and inhibiting inflammasome activation (*19*). Previously, we have reported that RNH1 is a ribosome-associated protein that regulates *GATA1* mRNA translation, and is responsible for defects in embryonic erythropoiesis in RNH1-deficient mice (*20*). In line with this, recent human molecular genetics studies have further revealed that mutations in *RNH1* gene lead to severe phenotypes such as global developmental delay, embryonic death, inflammation, and macrocytic anemia (*21*, *22*). Collectively, these findings strongly suggest that RNH1 is a multifunctional molecule crucial for mammalian survival (*23*). Here, we used a combination of human cell lines and mouse models, along with biochemical experiments, to establish the critical role of RNH1 in hematopoietic-specific translation. We found that RNH1, via its binding partner ANG, confers cell type–specific global translation regulation. Additionally, we discovered that RNH1 controls the translation of RP mRNAs and influences mRNA circularization. These findings provide valuable insights into the intricate regulatory mechanisms governing cell type–specific translation processes.

## RESULTS

### The loss of RNH1 predominantly reduces translation in hematopoietic-origin cells

**Fig. 1.**
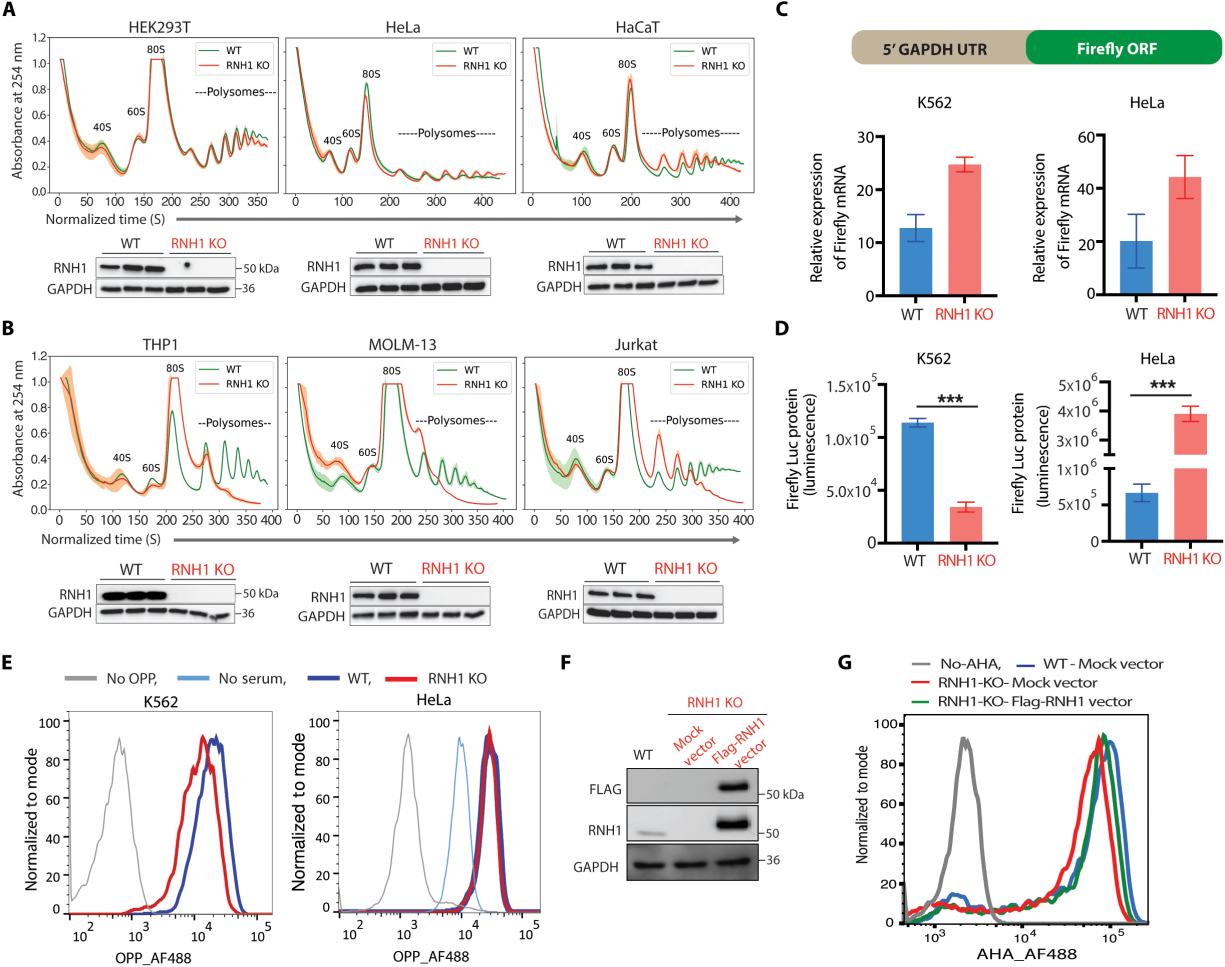
Loss of RNH1 decreases translation specifically in hematopoietic-origin cells. (**A** and **B**) Sucrose gradient polysome profiles for wild-type (WT) and corresponding RNH1 knockout (RNH1 KO) nonhematopoietic (A) and hematopoietic (B) origin cells (*N* = 3). Arrow shows the direction of the sucrose gradient from low to high density. Mean value of absorbance from three independent experiments plotted with the SD (upper panel). Total protein lysates of WT and RNH1 KO cells were analyzed by Western blot with the indicated antibodies. Blots are representative of three independent experiments (lower panel). (**C** and **D**) Schematics of luciferase-expressing plasmid with *GAPDH* 5′UTR (upper panel). WT and RNH1 KO HeLa and K562 cells were transfected with luciferase-expressing plasmid. Cells were analyzed for Firefly mRNAs by qRT-PCR and normalized to 18S rRNA expression (C) and luciferase protein expression by luciferase assay (D). Data are shown as means ± SEM and are representative of three independent experiments. ***P < 0.001. (**E**) WT or RNH1 KO K562 and HeLa cells were incubated for 1 hour with OPP, and fluorescence-activated cell sorting (FACS) analysis was performed to measure OPP incorporation. Representative histograms were shown for OPP fluorescence. Data are representative of three independent experiments. (**F** and **G**) RNH1 is ectopically expressed in RNH1 KO K562 cells. Cell lysates were analyzed by Western blot with the indicated antibodies. Blots are representative of three independent experiments (F). K562 cells were incubated for 1 hour with AHA, and FACS analysis was performed to measure AHA incorporation. Representative histograms were shown for AHA fluorescence. Data are representative of three independent experiments (G).

RNH1 is a ribosome-associated protein, and deletion of RNH1 in a human erythroleukemic cell line (K562 cells) has been shown to result in reduced polysomes levels (fig. S2A) (*20*). However, the detailed mechanism/s of RNH1-mediated translation regulation remains unknown. To investigate this, RNH1 was knocked out of human embryonic kidney (HEK) 293T cells, and the global translation status was checked by polysome analysis (Fig. 1A). Polysome levels were not altered in RNH1 knockout (KO) cells, suggesting that RNH1 might regulate cell type–specific translation. Next, RNH1 was knocked out in several randomly selected cell lines of different origins, and global translation status was checked by polysome analysis (Fig. 1, A and B). Cell lines from hematopoietic origin (THP1, MOLM13, and Jurkat) consistently showed a large reduction in polysome levels compared to their corresponding wild-type (WT) cells (Fig. 1B). However, polysome levels were not changed in RNH1 KO non–hematopoietic-origin cell lines such as HeLa, HaCaT, and SH-SY5Y compared to corresponding WT cells (Fig. 1A and fig. S2B). To our knowledge, such cell type–specific global translation defect has not been reported previously in mammalian cells. While mutations in RPs lead to cell type–specific functional defects (*4*), their knockdown dysregulates global translation in various cell types of different origin. For example, the *RPS19* gene is frequently mutated in DBA patients, and its knockdown leads to global translational defects in several cell types including both hematopoietic and non–hematopoietic-origin cells (HeLa, embryonic stem cells, different erythroid cell lines, and progenitor cells) (*6*, *7*, *24*–*27*). These results suggest that, unlike RP gene mutations, loss of RNH1 primarily leads to translation defects in hematopoietic-origin cells but not in non–hematopoietic-origin cells.

To confirm above polysome results, luciferase reporter assay was performed to check the translation. HeLa WT and RNH1 KO cells, as well as K562 WT and RNH1 KO cells, were transfected with a plasmid containing luciferase reporter gene having glyceraldehyde 3-phosphate dehydrogenase (*GAPDH*) 5′ untranslated region (UTR) (Fig. 1C). By maintaining same levels of luciferase mRNA in WT and RNH1 KO (Fig. 1C), we observed enhanced luciferase protein levels in HeLa RNH1 KO (Fig. 1D). On the contrary, K562 RNH1 KO cells showed a significant reduction in translation of luciferase reporter (Fig. 1D). Similar results were obtained with another luciferase reporter having β-actin (*ACTB*) 5′UTR (fig. S2C). Both *ACTB* and *GAPDH* 5′UTRs are simple UTRs without any secondary structure (*7*). Thus, this assay ruled out any contribution of the complexity of 5′UTRs in RNH1-mediated translation regulation. To further ascertain RNH1-mediated specific translation regulation, nascent polypeptide labeling via O-propargyl-puromycin (OPP) was performed. OPP is an analog of puromycin. It gets incorporated into newly translating proteins and can be detected by copper(I)-catalyzed azide-alkyne cycloaddition (CuAAC) (*28*). Supporting the above results, flow cytometry analysis showed reduced incorporation of OPP in K562 RNH1 KO cells, but not in HeLa RNH1 KO cells, compared to their respective WT cells (Fig. 1E). Furthermore, ectopic expression of RNH1 in RNH1 KO K562 cells was able to rescue the translation defects observed in K562 RNH1 KO (Fig. 1, F and G, and fig. S2D). To understand whether overexpression of RNH1 affects global translation, we generated stable RNH1-overexpressing K562 and HeLa cells and performed nascent polypeptide labeling with azidohomoalanine (AHA), an analog of methionine that incorporates into newly translating proteins (*29*). Supporting our previous study (*20*), overexpression of RNH1 increased global translation in K562 cell (fig. S2, E and G). However, in HeLa cells, overexpression of RNH1 did not increase translation (fig. S2, F and H). Altogether, these results confirm that loss of RNH1 reduces global translation only in cells of hematopoietic origin.

Differences in cell size and ribosomal gene expression between hematopoietic and non–hematopoietic-origin cell line might account for the observed translation defects (*30*). So, we checked cell size and ribosomal gene expression in these cell lines. These parameters varied across cell lines, and none of them clearly separated hematopoietic cells from non–hematopoietic-origin cell lines (fig. S3, A to C). Thus, cell size and ribosomal gene expression could not account for the RNH1-mediated global translation defects in hematopoietic cell lines. It is known that RNH1 interacts with ANG and inhibits its functions. Under stress situations, ANG mediates translation repression by degradation of tRNAs to form tRNA-derived stress-inducible RNAs (tiRNAs) in the cytoplasm (*17*). Therefore, we checked the possibility of increased tRNA degradation in RNH1 KO cells by assessing the levels of tiRNAs. We did not find an increase in tiRNAs in the RNH1-KO cells at steady state without stress conditions (fig. S3D). However, under stressed conditions, RNH1 KO K562 cells showed a minor increase in tiRNA production compared to WT (fig. S3D). This corroborates with a previous study where overexpression of ANG in rat neuronal cell line (PC12 cells) did not produce tiRNAs when cells were not stressed. However, under stress, ANG-overexpressing cells synthesized larger amounts of tiRNAs, even with mild stress (*31*). Thus, under steady-state situations, we observed a perturbation of translation in RNH1 KO hematopoietic cells, along with unchanged tiRNA levels. These results exclude involvement of tRNA degradation in RNH1-mediated cell type–specific translation regulation.

### RNH1 deficiency in mice specifically reduces the translation in hematopoietic cells

**Fig. 2.**
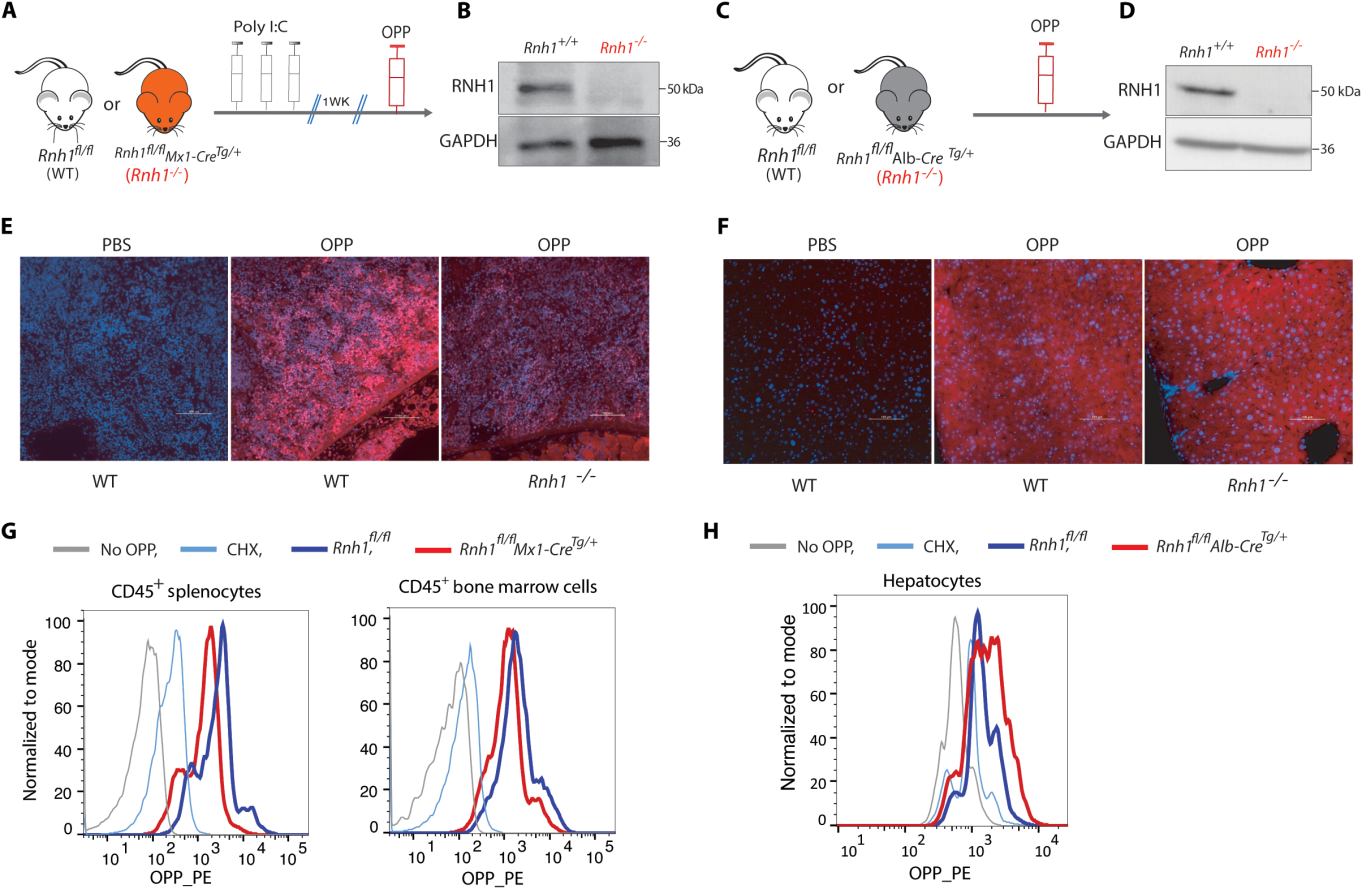
The absence of RNH1 specifically decreases translation in hematopoietic cells in mice. (**A** to **F**) OPP incorporation assay was performed after 1 hour of intraperitoneal injection with PBS or OPP (50 mg/kg) in WT (*Rnh1^fl/fl^*), hematopoietic-specific *Rnh1*-deficient (*Rnh1^fl/fl^, Mx1-Cre^+^*), and liver-specific *Rnh1*-deficient (*Rnh1^fl/fl^, Alb-Cre^+^*) mice. Schematics showing Mx1-Cre model, where *Rnh1* was excised by giving three rounds of 200 μg of poly(I:C) using intraperitoneal injections to *Rnh1^fl/fl^* (WT) and *Rnh1^fl/fl^Mx1-Cre^+^* (*Rnh1^−/−^*) mice. After 1 week, mice were injected with OPP and organs were harvested after 1 hour (A). Spleen cell lysates of WT and *Rnh1^−/−^* cells were analyzed by Western blot with the indicated antibodies. Blots are representative of three independent experiments (B). Schematics showing that *Rnh1^fl/fl^* (WT) and *Rnh1^fl/fl^ Alb-Cre^+^* (*Rnh1^−/−^*) mice were injected with OPP, and organs were harvested after 1 hour (C). Liver cell lysates of WT and *Rnh1^−/−^* cells were analyzed by Western blot with the indicated antibodies. Blots are representative of three independent experiments (D). OPP incorporated spleen (E) and liver (F) tissue sections were stained by CuAAC with TMR-azide and Hoechst (*n* = 3 mice for each genotype) (scale bar, 100 μm). (**G** and **H**) WT or *Rnh1^−/−^* splenocytes, bone marrow cells (G), and hepatocytes (H) were incubated for 1 hour with OPP, and FACS analysis was performed to measure OPP incorporation. Representative histograms were shown for OPP fluorescence (*n* = 2 mice for each genotype and each mice with three technical replicates).

To confirm the RNH1-mediated hematopoietic-specific translation regulation in vivo*,* OPP was injected into WT (*Rnh1^fl/fl^*), hematopoietic *Rnh1*-deficient (*Rnh1^fl/fl^, Mx-Cre^Tg/+^*) (Fig. 2, A and B), and liver-specific *Rnh1*-deficient (*Rnh1^fl/fl^, Alb-Cre^Tg/+^*) mice (Fig. 2, C and D). We refer *Rnh1*-deficient mice *as Rnh1^−/−^*. Corroborating our in vitro cell line experiments, the spleen of *Rnh1^−/−^* mice showed reduced incorporation of OPP compared to WT (Fig. 2E), indicating translation reduction. On the contrary, the liver of *Rnh1^−/−^* mice did not show translation repression (Fig. 2F). Further, OPP incorporation assay was performed using hematopoietic cells isolated from bone marrow and spleen, and hepatocytes from liver. In agreements with in vivo results, translation was decreased in mouse hematopoietic cells (Fig. 2G), but not in hepatocytes (Fig. 2H). Thus, using multiple complementary approaches, our results demonstrated that RNH1 specifically regulates global translation in hematopoietic cells in vivo and in vitro. These results also suggest that RNH1-mediated hematopoietic translation is species independent and occurs in both human and mice.

### RNH1 regulates translation of mRNAs encoding ribosomal proteins

**Fig. 3.**
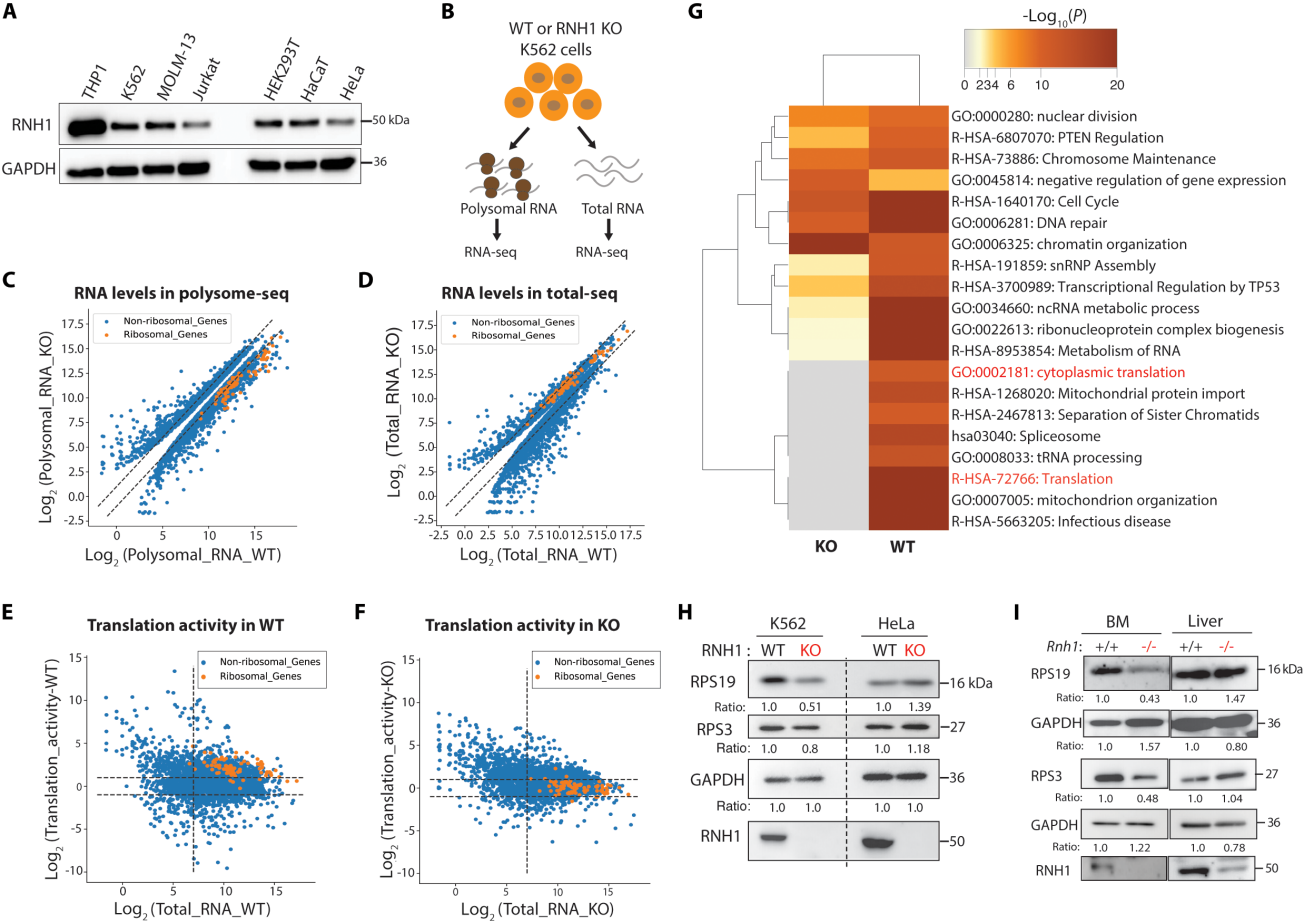
RNH1 is involved in ribosomal protein mRNA translation. (**A**) Total protein lysates from WT and RNH1 KO of hematopoietic and non–hematopoietic-origin cell lines were analyzed by Western blot with the indicated antibodies. Blots are representative of three independent experiments. (**B**) Schematic showing total and polysomal RNA isolation and RNA-seq analysis. (**C** and **D**) The expression of ribosomal and nonribosomal genes in WT and RNH1 KO K562 cells obtained by polysomal RNA-seq (C) or total RNA-seq (D). Total genes with cutoff of *P*_adj_ < 0.1 in polysomal RNA-seq and total RNA-seq were selected and plotted. Dotted lines separate genes with log_2_(fold change) <1 and >1. (**E** and **F**) Plot of log_2_(fold change) of total RNA versus translational activity (TA) of ribosomal and nonribosomal genes in WT (E) or RNH1 KO (F) samples. Horizontal dotted lines separate genes with log_2_(fold change) <1 and >1. Vertical lines separate genes with arbitrary cutoff of >7 or <7 for log_2_(expression in total RNA-seq). (**G**) The heat maps generated via Metascape tool with default parameters show the relative expression of genes in different gene ontology (GO) gene sets. (**H**) Protein lysates from K562 and HeLa with WT and RNH1 KO genotypes were analyzed by Western blot for RPS19, RPS3, and RNH1. Blots are representative of three independent experiments. GAPDH is used as a normalizing control, and numbers represent the normalized band intensity with respect to the corresponding WT sample. (**I**) Protein lysates from WT and *Rnh1^−/−^* bone marrow (BM) and liver were analyzed by Western blot for RPS19, RPS3, and RNH1 (*N* = 3 mice). GAPDH is used as a normalizing control, and numbers represent the normalized band intensity with respect to the corresponding WT sample.

To understand how RNH1 mediates hematopoietic-specific translation, we first examined its expression in different hematopoietic and nonhematopoietic cells lines. However, there was no major difference in RNH1 protein levels in both cell types that could explain the above observed translational defects (Fig. 3A). Next, to gain insights into the molecular clues involved in RNH1-mediated cell type–specific translation regulation, we performed polysome-associated RNA sequencing (polysome-seq) using polysomal RNA from K562 WT and RNH1 KO cells (Fig. 3B) and compared it with bulk RNA-seq (total-seq) data (table S1) (*20*). Polysome-seq analysis revealed that mRNAs encoding RPs were significantly less associated with polysomes in RNH1 KO K562 cells compared to WT cells (Fig. 3C), indicating reduced translation of RP mRNAs in RNH1 KO. On the contrary, total RNA-seq did not show any changes in the expression levels of RP mRNAs between WT and RNH1 KO cells (Fig. 3D). This suggests that the translation of RP mRNAs is perturbed in RNH1 KO hematopoietic cells without altering their mRNA levels. Next, translation activity (TA) was calculated as polysomal RNA/total RNA. As expected, RP mRNAs showed high expression and were more translationally active in WT cells (Fig. 3E). In RNH1 KO K562 cells, the TA of RP mRNAs was reduced without changes in RNA expression levels (Fig. 3F), confirming the translation repression of RP mRNAs in RNH1 KO cells. Additionally, we implemented analysis of translational activity (ANOTA), which applies per-gene analysis of partial variance with variance shrinkage (*32*). ANOTA identified a stringent set of 262 genes that were translationally deregulated in K562 RNH1 KO cells, including several RP mRNAs (table S2). This further confidently reiterates that the total RNA level of RP mRNAs is not changed in WT and KO K562 cells, but their polysome-associated RNA levels decreased significantly in RNH1 KO K562 cells (fig. S4, A and B). TA of genes that retained after ANOTA analysis also showed that WT cells have high expression of RP mRNAs and large TA, while in RNH1 KO, TA of RP mRNAs decreased without change in their RNA levels (fig. S4, C and D). We selected genes with high expression and large TA from WT (1549) and KO (884) (genes in the upper right quadrant of plots in Fig. 3, E and F) and performed gene ontology (GO) analysis. The enrichment of GO terms in WT and KO showed that terms like “translation” and “cytoplasmic translation” were significantly less represented in RNH1 KO compared to WT (Fig. 3G). This further suggests that RNH1 KO K562 cells have reduced TA. To confirm this, cell lysates of K562 and HeLa with WT and RNH1 KO genotypes were analyzed by Western blots for representative RPs. As expected, K562 RNH1 KO cells showed reduced levels of RPS19 and RPS3 proteins compared to K562 WT cells. On the contrary, RPS19 and RPS3 levels in HeLa RNH1 KO cells did not decrease (Fig. 3H). Similarly, RPS19 and RPS3 levels were reduced in *Rnh1^−/−^* mice bone marrow cells but not in the liver compared to WT control mice (Fig. 3I). These results suggest that RNH1 is involved in RP gene translation.

**Fig. 4.**
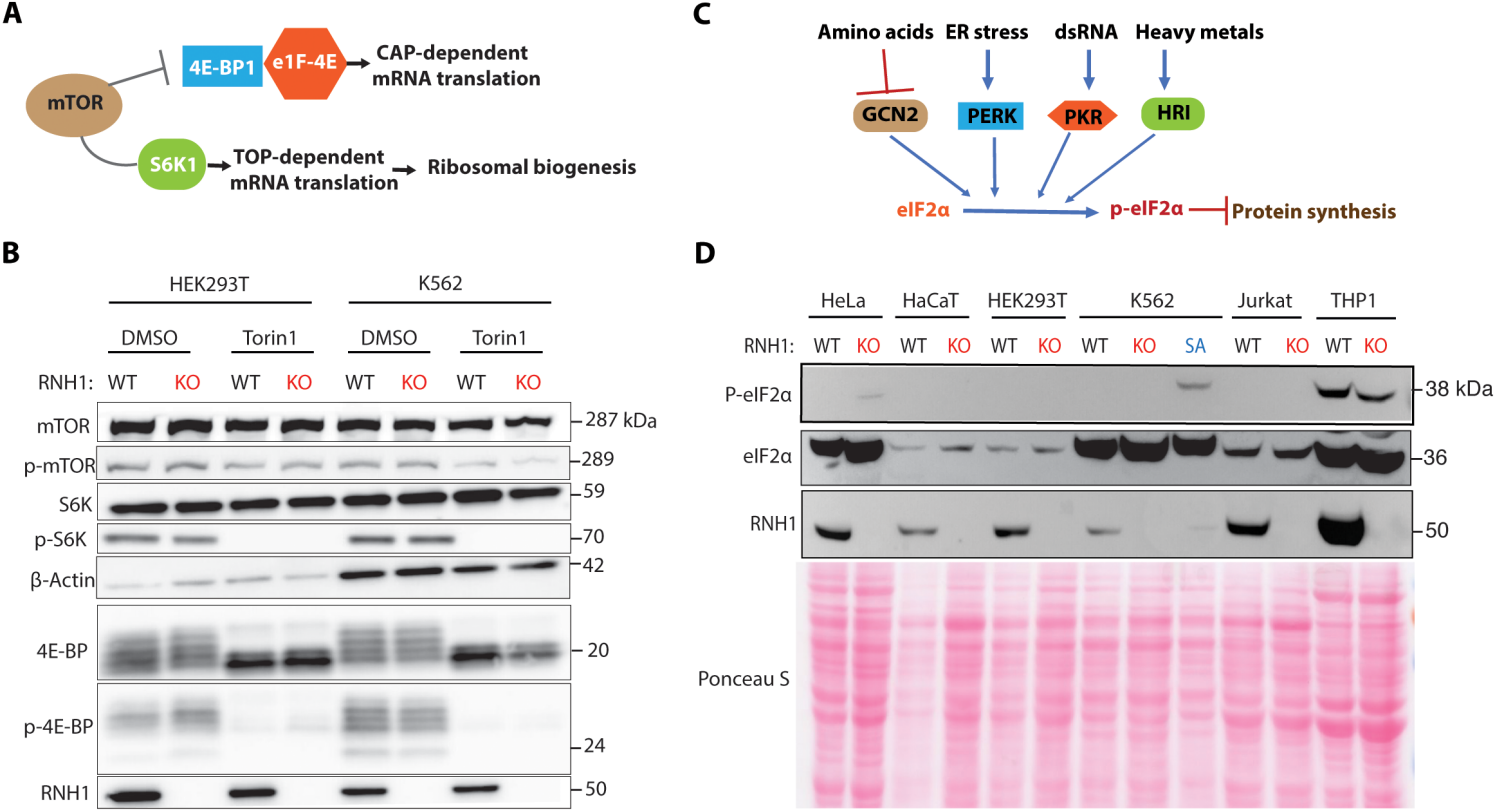
Unaltered mTOR signaling and phosphorylation of eIF2α in the absence of RNH1. (**A**) Schematic of mTOR signaling. (**B**) WT and RNH1 KO HEK293T and K562 cells were treated with or without mTOR inhibitor Torin1 (100 nM) and analyzed by Western blot with the indicated antibodies. Blots are representative of four independent experiments. (**C**) Schematic of eIF2α kinases mediated translation suppression. (**D**) WT and RNH1 KO HeLa, HaCaT, HEK293T, K562, Jurkat, and THP1 cell lysates were analyzed by Western blot with the indicated antibodies. Blots presented are representative of two independent experiments. K562 cells treated with sodium arsenate (SA) (100 μM) are used as positive control for stress-induced translation inhibition.

To comprehend the mechanism by which RNH1 regulates RP mRNA translation, we investigated mammalian target of rapamycin (mTOR) signaling in RNH1 KO and WT HEK293T and K562 cell lines. The mTOR pathway is known to regulate RP mRNA translation and global translation after nutrient sensing (Fig. 4A) (*33*). However, we did not observe differences in the phosphorylation levels of mTOR, S6K, and 4E-BP between WT and RNH1 KO cells in both cell types (Fig. 4B). The mTOR pathway regulates RP mRNA translation through 5′ terminal oligopyrimidine (TOP) motifs (*33*), so we tested mRNA reporter system with and without TOP motifs to determine how loss or overexpression of RNH1 affects their translation. We transfected K562 cells with Renilla reporter constructs containing the promoter and 5′UTRs of eukaryotic elongation factor 2 (eEF2) with either WT or mutant TOP sequences (*33*). The loss of RNH1 decreased the translation of both TOP and mutant TOP constructs (fig. S5A), Further, overexpression of RNH1 partially increased TOP translation but not mutant TOP translation (fig. S5B). These data suggest that RNH1 regulates RP mRNA translation independent of mTOR signaling. Another important factor controlling global translation is eukaryotic initiation factor 2α (eIF2α). Phosphorylation of eIF2α after cellular stress, mediated by eIF2α kinases (GCN2, PERK, HRI, and PKR), inhibits global translation and potentially contributes to the decrease in RP mRNA translation (Fig. 4C) (*34*). However, we did not observe differences in the phosphorylation levels of eIF2α between WT and RNH1 KO cells in both cell types (Fig. 4D). This excludes the possibility of stress-mediated translational defects in RNH1 KO hematopoietic cells. Collectively, these results indicate that mTOR signaling and eIF2α phosphorylation, which are two major pathways involved in global translation regulation (*35*), do not account for the translational defects mediated by loss of RNH1 in hematopoietic cells.

### RNH1 binds to PABP and is involved in mRNA circularization

**Fig. 5.**
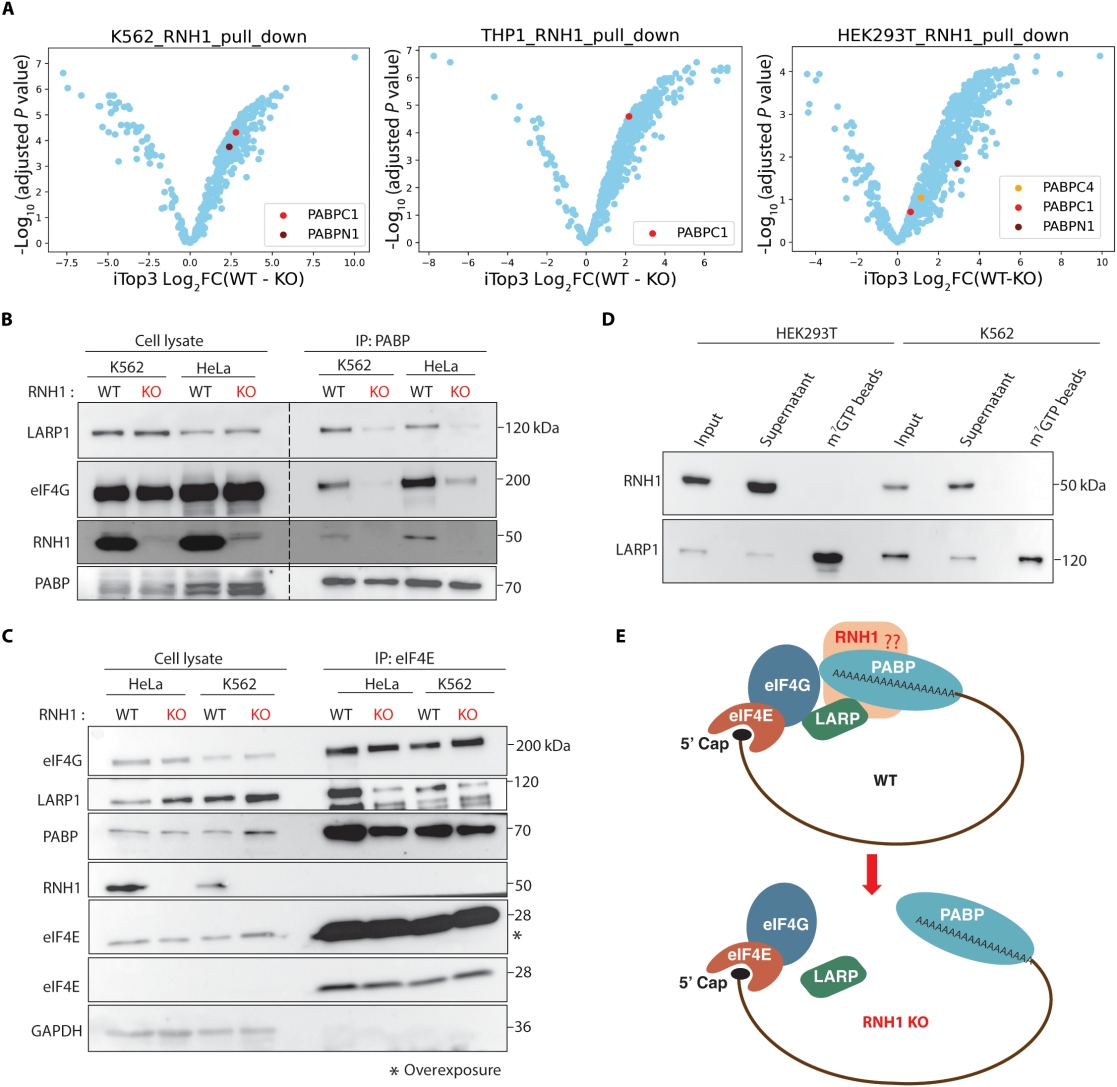
RNH1 binds to PABP and helps in mRNA circularization. (**A**) Volcano plots depicting RNH1 binding total proteome from WT K562, THP1, and HEK293T cells compared with RNH1 KO of corresponding cells. Plots show the comparisons of log_2_(fold changes) (FC) versus adjusted *P* values (false discovery rate–controlled Benjamini and Hochberg multiple test correction) calculated based on peptide-imputed Top3 (iTop3). (**B** and **C**) Whole-cell lysates of WT and RNH1 KO K562 and HeLa cells were used for anti–PABP-IP (B) and anti–eIF4E-IP (C) and then immunoblotted, as indicated. Blots are representative of three independent experiments. (**D**) Whole-cell lysates of K562 and HEK293T cells were used for m^7^GTP IP and immunoblotting, as indicated. Blots are representative of three independent experiments. (**E**) Schematics of RNH1-mediated mRNA circularization.

Our previously published mass spectrometry (MS) experiments have revealed that RNH1 directly binds to RPs and several RNA processing proteins (*20*). Among its interacting partners, poly(A) binding protein (PABP) caught our attention. Recently, another study also confirmed the binding of RNH1 to PABP (*36*). To further investigate these interactions, we performed co-immunoprecipitation (IP) of endogenous RNH1-interacting proteins using different cell lines (THP1, K562, and HEK293T) followed by MS (fig. S6, A and B, and tables S3 to S5). Supporting previous results, PABP was found to bind to RNH1 in all three cell lines (Fig. 5A). PABP is a well-known translation initiation factor that binds to the poly(A) tail of mRNAs (*37*). Its interaction with poly(A) tail stimulates translation by associating it with the eukaryotic initiation factor 4G (eIF4G) (*38*). This interaction helps to circularize mRNAs by bringing the 3′ end close to its 5′ m^7^G cap via sequential interactions of the 3′-poly(A)-PABP-eIF4G-eIF4E-5′ m^7^G cap (*37*). These interactions play pivotal roles in the translation initiation and maintaining translation efficiency in eukaryotic cells (*39*). Transcripts with a TOP sequences, such as RPs, could be hypersensitive to defects in these interactions (*40*). To further check whether the RNH1 and PABPC1 interactions are poly(A) tail dependent, we performed isothermal titration calorimetry (ITC) using full-length recombinant RNH1 protein and PABPC1-A_24_ [A_24_: 24-mer poly(A), the length of poly(A) covering one molecule of PABPC1] (*41*). No change in heat quantity was detected through the injections, indicating that RNH1 did not bind to PABPC1-A_24_ under this condition (fig. S7A). The spikes detected at regular intervals are due to heat of dilution of the sample and not due to binding of RNH1 to PABPC1-A_24_. To ascertain quality of purified PABPC1, i.e., whether PABPC1 has the ability of binding to poly(A), we tried the interaction analysis of PABP and A_24_. Changes in heat quantity were detected, indicating that PABP bound to A_24_ (fig. S7B). However, the absence of the interaction between RNH1 and PABPC1 in the ITC experiments, despite previous observations (*36*) and the aforementioned MS experiments (Fig. 5A), suggests that other protein(s) might be involved in mediating the binding of RNH1 with PABPC1 endogenously.

To check the role of RNH1 in mRNA circularization, PABP-interacting partners from WT and RNH1 KO K562 cells were coimmunoprecipitated. As expected, eIF4G and RNH1 were immunoprecipitated in the PABP-IP of WT K562 cells (Fig. 5B). PABP and eIF4G interactions were decreased in RNH1 KO K562 cells (Fig. 5B). This reduction in interaction between eIF4G⇔PABP might have prevented mRNA circularization and led to the observed translation decrease in RNH1 KO hematopoietic cells. La-related protein 1 (LARP1) is another interacting partner of PABP involved in the translation of RP mRNAs (*42*). LARP1 and PABP interaction also decreased in RNH1 KO K562 cells (Fig. 5B). The levels of PABP, eIF4G, or LARP1 were not altered in WT and RNH1 KO K562 total cell lysates (Fig. 5B). This suggests the dysregulated assembly of factors at the 3′ end of mRNAs in the RNH1 KO K562 cells. To check the scenario at the 5′ end of mRNAs, we pulled down eIF4E-interacting partners, as eIF4E is a cap-binding protein. eIF4E-IP revealed that eIF4E-eIF4G interactions were not altered, but LARP1 and PABP interactions were reduced in RNH1 KO eIF4E-IP (Fig. 5C). This hints that assembly of eIF4E and eIF4G on 5′ cap is unaltered in RNH1 KO cells. LARP1 and PABP were immunoprecipitated with eIF4E likely due to secondary interactions, as there are no direct interactions between LARP1⇔eIF4E⇔PABP known to our knowledge. Further, m^7^GTP cap-binding assay revealed that RNH1 does not bind to the 5′ cap in both hematopoietic and nonhematopoietic cells (Fig. 5D). Thus, RNH1 is absent at the 5′ end and present at the 3′ end of mRNAs via PABP and other unknown factors, supporting reduced interactions between eIF4G⇔PABP but not between eIF4E⇔eIF4G (Fig. 5, B and C). Thus, loss of RNH1 affects mRNA circularization and/or translation initiation by regulating LARP1⇔PABP⇔eIF4G interactions (Fig. 5E). To our surprise, LARP1⇔PABP⇔eIF4G interactions were also reduced in nonhematopoietic RNH1 KO HeLa cells, suggesting that RNH1 regulates LARP1⇔PABP⇔eIF4G interactions in both hematopoietic and nonhematopoietic cells (Fig. 5, B and C). However, this raises the question of why the loss of RNH1 predominantly reduces translation in hematopoietic-origin cells but not in non–hematopoietic-origin cells.

### ANG compensates loss of RNH1-mediated translation defects in nonhematopoietic cells

**Fig. 6.**
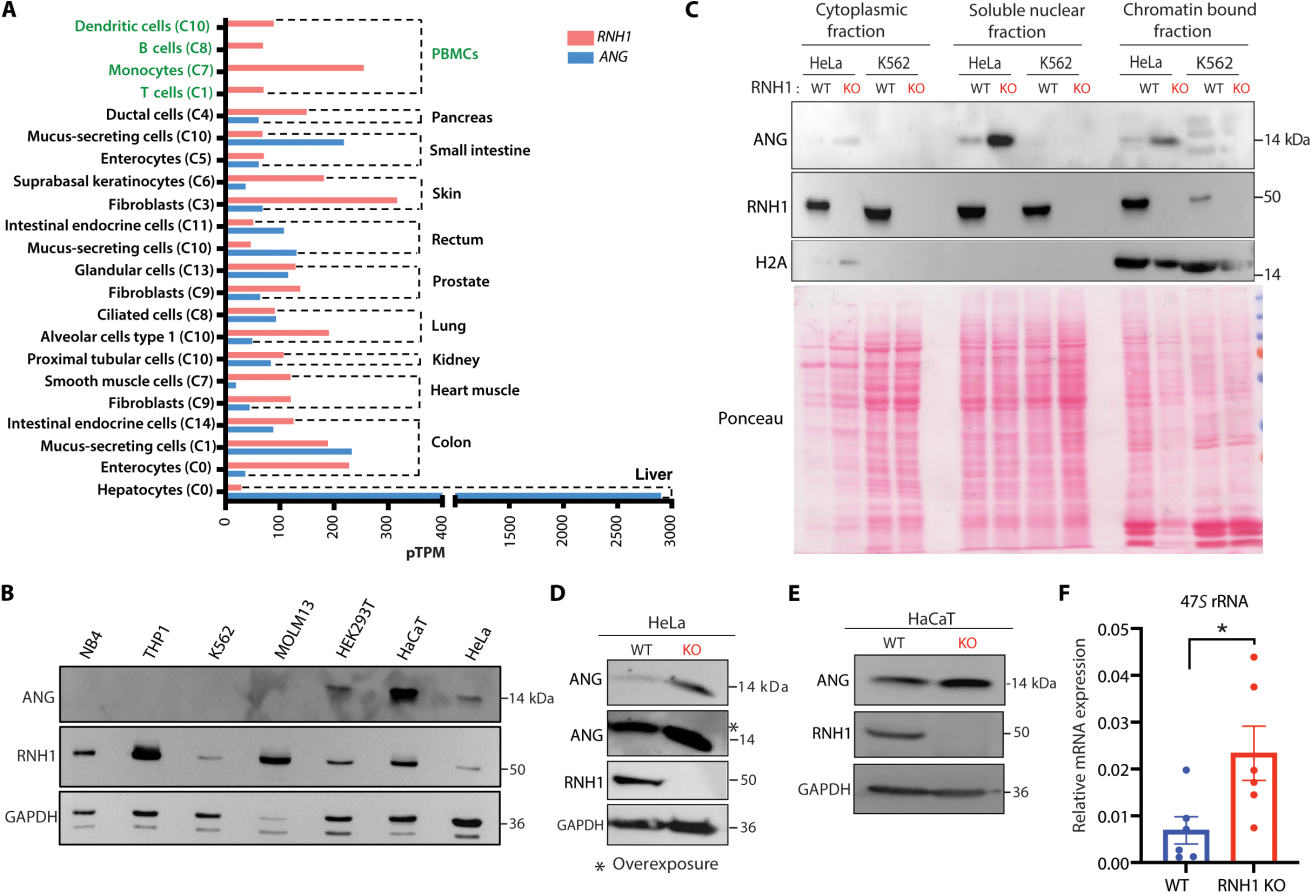
RNH1 deficiency increases ANG expression in nonhematopoietic cells. (**A**) RNH1 and ANG mRNA expression from single-cell RNA sequencing of different human cells from hematopoietic and nonhematopoietic origin. C0 to C11 are cluster numbers. pTPM, protein-transcripts per million. Data source: https://www.proteinatlas.org. (**B**) Total protein lysates from hematopoietic- and non–hematopoietic-origin cell lines were analyzed by Western blot with the indicated antibodies. Blots are representative of three independent experiments. (**C**) Total protein lysates of different cellular fractions from WT and RNH1 KO of K562 and HeLa cells were analyzed by Western blot with the indicated antibodies. Blots are representative of three independent experiments. (**D** and **E**) Total protein lysates from WT and RNH1 KO HeLa (D) or HaCaT (E) cells were analyzed by Western blot with the indicated antibodies. Blots are representative of three independent experiments. (**F**) qRT-PCR analysis of WT and RNH1 KO HeLa cells for 47S rRNA, normalized to 18S rRNA. Data are shown as means ± SEM and are representative of six independent experiments. *P < 0.05.

To address the aforementioned discrepancy, we formulated a hypothesis that non–hematopoietic-origin cells might have specific compensatory mechanisms enabling them to tolerate the RNH1 loss. To explore potential candidates for further investigation, we sought out translation regulators that interact with RNH1 and are exclusively expressed in nonhematopoietic cells but not in hematopoietic-origin cells. ANG emerged as a promising candidate, meeting all the criteria. RNH1 is known to bind to ANG and inhibit its functional activities (*12*). ANG exhibits a very weak ribonucleolytic activity but has strong capabilities in inducing blood vessel growth. It has been implicated in the initiation, progression, and metastasis of tumors (*12*, *43*). Furthermore, ANG plays contrasting roles in translation (*13*, *44*). In stress situations, it cleaves tRNA and decreases translation (*45*). Under favorable growth conditions, ANG translocates to the nucleus, triggering the synthesis of ribosomal RNA (rRNA) and actively participating in cell proliferation (*46*, *47*). To investigate the expression pattern of ANG in hematopoietic- and non–hematopoietic-origin primary human cells, we analyzed publicly available single-cell RNA-seq datasets. We found minimal to no expression of ANG in hematopoietic cells compared to nonhematopoietic cells (Fig. 6A). In line with this, we did not find ANG protein expression in hematopoietic-origin cell lines (Fig. 6B). This finding suggests that ANG might play a distinct role in translation regulation in nonhematopoietic cells, potentially contributing to their ability to cope with the loss of RNH1. The presence of ANG in the nucleus enhances rRNA synthesis (*46*), so we checked its localization by analyzing subcellular fractions of WT and RNH1 KO HeLa and K562 cells. As expected, ANG was not detected in both WT and RNH1 KO K562 cells (Fig. 6C). In RNH1 KO HeLa cells, ANG expression increased in the nucleus and also other cell fractions compared to WT HeLa cells (Fig. 6C). Corroborating this, ANG levels were also increased in RNH1 KO HeLa and HaCaT total cell lysates (Fig. 6, D and E). These results suggest that ANG is either expressed minimally or not at all in hemopoietic-origin cells, but is expressed in non–hematopoietic-origin cells. Additionally, the loss of RNH1 leads to increase in ANG expression and its translocation into the nucleus of non–hematopoietic-origin cells. To check whether the increased nuclear ANG enhances rRNA transcription in RNH1 KO cells, we quantified 47*S* rRNA levels in RNH1 KO HeLa cells via quantitative polymerase chain reaction (qPCR). We found increased rRNA transcription in RNH1 KO HeLa cells (Fig. 6F), supporting the notion that ANG plays a compensatory role in these cells. These results indicate that ANG rescues the loss of RNH1-mediated translation defects in nonhematopoietic cells. This could explain the increased translation observed in RNH1 KO HeLa cells in luciferase reporter assay (Fig. 1D) as well as the elevated RP expression (Fig. 3H).

**Fig. 7.**
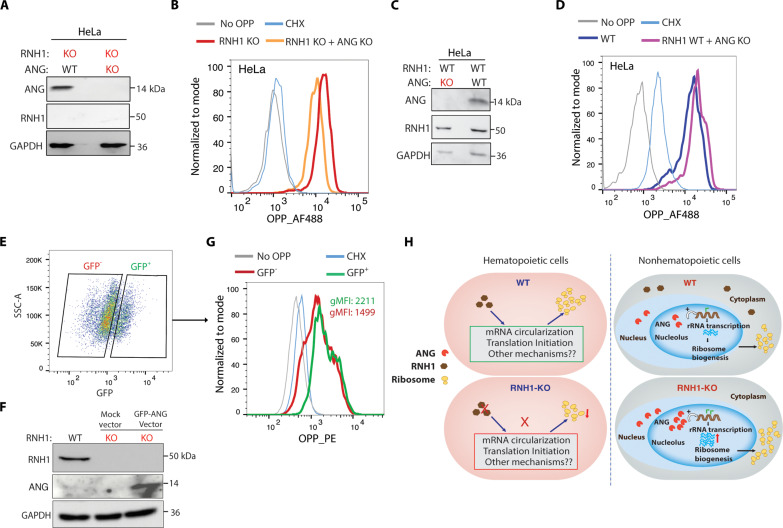
ANG compensates for translational defects in RNH1-deficient nonhematopoietic cells. (**A**) Total protein lysates from RNH1 KO or RNH1 and ANG double KO HeLa cells were analyzed by Western blot with the indicated antibodies. Blots are representative of three independent experiments. (**B**) RNH1 KO or RNH1 and ANG double KO HeLa cells were incubated for 1 hour with OPP, and FACS analysis was performed to measure OPP incorporation. Representative histograms were shown for OPP fluorescence. Data are representative of three independent experiments. (**C**) Total protein lysates from WT or ANG KO HeLa cells were analyzed by Western blot with the indicated antibodies. Blots are representative of three independent experiments. (**D**) WT or ANG KO HeLa cells were incubated for 1 hour with OPP, and FACS analysis was performed to measure OPP incorporation. Representative histograms were shown for OPP fluorescence. Data are representative of three independent experiments. (**E** to **G**) RNH1 KO K562 cells were transduced with GFP-tagged ANG and incubated for 1 hour with OPP, and FACS analysis was performed to measure OPP incorporation. FACS analysis shows GFP^+^ and GFP^−^ expressing cells. Data are representative of three independent experiments (E). Total protein lysates from control or GFP-tagged ANG-transduced RNH1 KO K562 cells were analyzed by Western blot with the indicated antibodies. Blots are representative of three independent experiments (F). FACS analysis was performed to measure OPP incorporation in ANG-GFP–transduced RNH1 KO K562 cells. Representative histograms were shown for OPP fluorescence (G). Data are representative of three independent experiments. gMFI, geometric mean fluorescence intensity. (**H**) Illustration of proposed RNH1/ANG–mediated cell type–specific translational regulation.

To further ascertain that ANG mediates compensatory mechanism, we deleted ANG in RNH1 KO HeLa cells and monitored translation by OPP incorporation (Fig. 7A). As hypothesized, loss of ANG decreased translation in RNH1 KO HeLa cells (Fig. 7B), while ANG deletion in WT HeLa cells had no impact on translation (Fig. 7, C and D). These results indicate that the loss of ANG has no impact on translation under steady-state conditions. Additionally, we investigated whether expressing ANG in RNH1 KO K562 cells could rescue translational defects. We ectopically expressed GFP-tagged ANG in RNH1 KO K562 cells (Fig. 7, E and F) and monitored translation by OPP incorporation. Supporting the above results, ANG-expressing RNH1 KO K562 cells (GFP^+^) showed increased translation compared to RNH1 KO K562 cells without ANG expression (GFP^−^) (Fig. 7G). Collectively, these results suggest that ANG-mediated translation compensates for the translation defects caused by the loss of RNH1 in nonhematopoietic cells (Fig. 7H). This compensatory mechanism involving ANG could be crucial for maintaining translation efficiency and cellular functions in the absence of RNH1.

We next asked whether RNH1-mediated cell type–specific translational defects result in tissue-specific functional impairments. Our previous findings demonstrated that the loss of RNH1 leads to defects in embryonic erythropoiesis. Further, knockdown of RNH1 in human CD34^+^ cells decreased erythroid differentiation (*20*). Similarly, deletion of RNH1 in mouse hematopoietic cells was found to dysregulate hematopoiesis and cause anemia (*48*). Moreover, mutations in the *RNH1* gene in human have also been associated with anemia phenotype (*21*). Overall, these studies collectively provide compelling evidence that the loss of RNH1 leads to defects or dysregulation in hematopoiesis. In contrast to the hematopoietic system, we observed no major phenotypic differences in liver-specific *Rnh1^−/−^* (*Rnh1^fl/fl^, Alb-Cre^Tg/+^*) mice compared to WT. Liver-specific *Rnh1^−/−^* mice exhibited normal survival rates, and there were no changes in body as well as liver weight (fig. S8, A to C). Additionally, liver function tests, such as alanine transaminase (ALT) and aspartate transaminase (AST) assays, also showed no significant differences between WT and liver-specific *Rnh1^−/−^* mice (fig. S8, D and E). Furthermore, blood glucose and peripheral blood levels were also similar in both groups (fig. S8, F and G). Moreover, we analyzed these mice for 18 months and did not notice any survival differences between WT and liver-specific *Rnh1^−/−^* mice (fig. S8H). Although we cannot completely exclude the possibility of translation-independent RNH1 functions contributing to tissue-specific phenotype, the absence of major phenotypic differences in liver-specific *Rnh1^−/−^* mice strongly indicate that the absence of RNH1 leads to tissue-specific defects, consistent with the observed translation defects in specific tissues.

## DISCUSSION

Collectively, these findings unveil the existence of cell type–specific global translation regulation, with RNH1, a ribosome-associated protein, emerging as a key regulator of translation. Our results suggest that RNH1 potentially governs the global translation by controlling the translation of ribosomal protein transcripts by regulating mRNA circularization. Nevertheless, further investigations are required to fully comprehend the precise mechanisms by which RNH1 mediates translation regulation. Consistent with the role of RNH1 in translation, we observed a decrease in translation upon the loss of RNH1 in hematopoietic-origin cells. However, this decrease in translation was not observed in non–hematopoietic-origin cells. Our results conclusively demonstrate that this translation specificity is mediated by a compensatory mechanism regulated by ANG. In non–hematopoietic-origin cells, ANG is expressed, and the loss of RNH1 further amplifies its expression. This increased ANG expression enhances translation through rRNA synthesis and compensates for the translation defects caused by RNH1 loss. Conversely, hematopoietic-origin cells either do not express ANG or express it at very low levels, making them susceptible to translational defects following RNH1 loss (Fig. 7H). However, how ANG-mediated increase of rRNA production overcomes mRNA circularization defects and increases global translation in RNH1-deficient non–hematopoietic-origin cells requires further studies. While our results strongly indicate that the loss of RNH1 substantially reduces translation in hematopoietic-origin cells, we cannot dismiss the possibility that other cell types might also be vulnerable to RNH1-mediated translation defects, considering the existence of over 200 different cell types in the human body (*49*).

Genes involved in translation, such as ribosomal protein genes, are highly conserved across eukaryotes. However, RNH1 and ANG genes have only evolved in vertebrates (fig. S1) (*50*), suggesting that vertebrates have developed an additional layer of translation control through cell type–specific regulation. These regulatory or compensatory mechanisms may play a crucial role in facilitating efficient protein translation and ensuring overall organismal survival. Although further investigation is warranted, it is tempting to speculate that these compensatory mechanisms might contribute to the specific phenotypes observed in ribosomopathies. Our study serves as a prime example of the regulation of cell type–specific global translation, highlighting the significance of comprehending these regulatory mechanisms to gain a comprehensive understanding of the translation process in vertebrates. Considering the substantial role of translation dysregulation in cancer progression, neurological diseases, and ribosomopathies (*4*, *35*, *51*, *52*), understanding tissue-specific translation regulators would be highly advantageous for targeting these specific diseases.

## MATERIALS AND METHODS

### Generation of mouse model and in vivo OPP incorporation studies

Generation of *Rnh1* conditional KO (*Rnh1^fl/fl^*) mice was reported previously (*19*). We crossed *Rnh1^fl/fl^* mice with *Mx1-Cre* (Jackson: 002527) and Albumin Cre mice (Jackson: 0103574) to generate inducible *Rnh1^−/−^* mice (*Rnh1^fl/fl^ Mx1-Cre*^+^) and liver-specific *Rnh1^−/−^* mice (*Rnh1^fl/fl^, Alb-Cre^Tg/+^*), respectively. *Rnh1* was excised in *Rnh1^fl/fl^ Mx1-Cre*^+^ mouse model by giving three rounds of 200 μg of Polyinosinic:polycytidylic acid [poly(I:C)] (InvivoGen) using intraperitoneal injections once every 2 days. The screening for *Rnh1* deletion after Cre recombination was performed by PCR genotyping as described previously (*19*). Western blot was also performed using spleen and liver to check RNH1 protein expression. The genotypes of the mice could not be blinded or randomized due to the experimental design. All groups of mice (experimental and control mice) were age- and sex-matched. We used littermates for our experiments. We used mice aged 8 to 12 weeks for experiments, unless otherwise stated. Mice were maintained at specific pathogen–free (SPF) conditions at the University Bern, Switzerland. All animal experiments were approved by the Swiss Federal Veterinary Office (Bern, Switzerland) under valid authorization (BE19/2019, BE103/2022). Mice were handled according to Swiss Federal Veterinary Office guidelines under valid authorization.

*Rnh1^−/−^* mice (*Rnh1^fl/fl^ Mx1-Cre*^+^ and *Rnh1^fl/fl^, Alb-Cre^Tg/+^* mice) and WT littermates (*Rnh1^fl/fl^ Cre*^−^) were injected intraperitoneally with OPP (Invitrogen) (50 mg/kg). Phosphate-buffered saline (PBS) was injected in the vehicle group. After 1 hour, the organs were collected and embedded in paraffin. Staining was performed as described previously (*28*). Briefly, after fixation and permeabilization, tissue slides were stained by CuAAC with the tetramethylrhodamine (TMR)–azide (Click-iT reaction) to fluorescently label OPP incorporated peptides. Finally, the tissue sections were counterstained with Hoechst, mounted in standard mounting media, and then imaged using a Nikon Ti-E fluorescence microscope.

### Cells and cell culture media

Cell lines K562, HEK293T, THP1, MOLM13, Jurkat, HeLa, HaCaT, and SH-SY5Y were grown in RPMI 1640 GlutaMAX-I medium (Invitrogen) or DMEM (500 ml, Thermo Fisher Scientific) supplemented with 10% (v/v) fetal bovine serum (FBS) (Amimed) and 1% penicillin and streptomycin (PAA Laboratories) and incubated at 37°C and 5% CO_2_. K562, HEK293T, THP1, Jurkat, HeLa, and HaCaT cell lines were described previously (*20*, *53*, *54*). Cell lines were tested negative for mycoplasma contamination using the MycoAlert Mycoplasma Detection Kit (Lonza, catalog no. LT07-318). MOLM13 cells were purchased from the American Type Culture Collection. Jurkat and HeLa cells were provided by P. Schneider (University of Lausanne, Lausanne, Switzerland). HaCaT cells were provided by E. J. Müller (University of Bern, Bern, Switzerland). SH-SY5Y cells were provided by S. Saxena (University of Bern, Bern, Switzerland).

### Reagents and plasmids

We purchased cycloheximide (CHX) and sodium arsenite from Sigma-Aldrich, mTOR inhibitor Torin-1 from InvivoGen, and Click-iT reaction kit and Dynabeads from Invitrogen (Thermo Fisher Scientific). Full-length RNH1-Flag construct and full-length GFP-RNH1 plasmids were described previously (*20*). To generate full-length GFP-ANG plasmid, the human ANG full coding sequence (ORIGEN RC 208874) was cloned into the entry vector pENTR4-GFP-C1 (W392-1, Addgene) and then recombined into the destination vector pLenti CMV Blast DEST (706-1, Addgene) plasmid using the Gateway cloning method. We purchased Renilla reporter constructs containing the promoter and 5′UTRs of eEF2 with either WT (*pIS1-Eef25UTR-renilla*; 38235) or mutant TOP sequences (*pIS1-Eef25UTR-TOPmut-renilla*; 38236) from Addgene.

### Generation of CRISPR/Cas9-mediated RNH1 and ANG KO cells

RNH1 KO cell lines were generated as described previously (*20*). For generation of ANG KO in WT and RNH1-KO HeLa cells, CRISPR guide RNA (gRNA) sequences targeting exon 1 on different regions of human ANG were designed and KO cells were obtained as described previously (*20*). Target exon and the seed sequences preceding the protospacer adjacent motif (PAM) are as follows: ANG_sgRNA1_F, 5′-CACCGAACGTTTCTGAACCCCGCTGTGG-3′; ANG_sgRNA1_R, 5′-AAACCCACAGCGGTTCAGAAACGTTC-3′. Primer2: ANG_sgRNA2_F, 5′-CACCGTGGCAACAAGCGCAGCATCAAGG-3′; ANG_sgRNA2_R, 5′-AAACCCTTGATGCTGCGCTTGTTGCCAC-3′. All generated KO clones were tested negative for mycoplasma contamination using the MycoAlert Mycoplasma Detection Kit (Lonza, catalog no. LT07-318).

### Reconstitution of Flag-RNH1 in RNH1-KO K562 cells

Flag-RNH1 was subcloned into the retroviral vector pMSCVpuro (Clontech) as described previously (*20*). Retroviral vector pMSCVpuro-Flag–RNH1 was cotransfected with the helper plasmids VSV-G and Hit60 into HEK293T cells using PEI transfection reagent. Culture supernatants containing recombinant viral particles were harvested and used to infect RNH1-KO K562 cells. To establish stable cell lines, puromycin (5 μg/ml) was added to the cells 3 days after infection for selection.

### Generation of RNH1-KO K562 cells expressing GFP-ANG

RNH1-KO K562 cells were infected with lentiviruses expressing ANG (pLenti CMV Blast GFP-ANG) or the empty vector pLenti CMV Blast DEST (706-1, Addgene) as previously described (*55*). At 48 hours after infection, cells were used for OPP incorporation experiments.

### Generation of Flag-RNH1–overexpressing K562 and HeLa cells

Retroviral vector pMSCVpuro-Flag–RNH1 was cotransfected with the helper plasmids VSV-G and Hit60 into HEK293T cells using PEI transfection reagent. Culture supernatants containing recombinant viral particles were harvested and used to infect K562 and HeLa cells. To establish stable cell lines, puromycin (5 μg/ml) was added to the cells 3 days after infection for selection.

### In vitro OPP and AHA incorporation assays

To measure the translation in cells, we performed OPP incorporation assays. OPP is an analog of puromycin that gets incorporated into nascent polypeptides. Cells were incubated with OPP (50 μM) for 1 hour and then washed with PBS. Click-iT reaction was performed using Alexa Fluor 488–azide or TAMRA-azide and the Click-iT Protein Reaction Buffer Kit (C10276, Invitrogen) for 30 min according to the manufacturer’s protocol.

For cell lines resistant to puromycin, we used AHA instead of OPP. To deplete methionine reserves, cells were incubated for an hour in a methionine-free RPMI medium (R7513, Sigma-Aldrich) containing 2 mM l-glutamine. Then, cells were washed and treated with AHA (C10102, Life Technologies) at a final concentration of 50 μM for 1 hour. After incubation, cells were washed with PBS, and Click-iT reaction was performed using Alexa Fluor 488–alkyne or TAMRA-alkyne and the Click-iT Protein Reaction Buffer Kit (C10276, Invitrogen) for 30 min according to the manufacturer’s protocol.

### Hepatocyte isolation

Hepatocytes were isolated from WT (*Rnh1^fl/fl^*) and *Rnh1^−/−^* mice (*Rnh1^fl/fl^, Alb-Cre^Tg/+^*) by using a two-step collagenase perfusion method, as previously described (*56*). The isolated live hepatocytes were plated and subjected to OPP incorporation assays as described above.

### Luciferase reporter assays

WT or RNH1 KO cells were transfected with plasmid containing luciferase reporter gene having *ACTB* or *GAPDH* 5′UTR (*7*). Lipofectamine 3000 was used for transfection as per the manufacturer’s instructions. After 48 hours of incubation, cells were washed and lysed in a lysis buffer (E1500 Promega). Lysates were mixed with Firefly luciferase assay reagent (E1500 Promega) as per the manufacturer’s protocol and scanned for luminescence in a Tecan scanner.

### Renella luciferase reporter assays

WT or RNH1 KO K562 cells we transfected with Renilla reporter constructs containing the promoter and 5′UTRs of eEF2 with either WT or mutant TOP sequences (*33*). Lipofectamine 3000 was used for transfection as per the manufacturer’s instructions. After 24 hours of incubation, cells were mixed with Renilla-Glo luciferase assay reagent (E2710, Promega) as per the manufacturer’s protocol and scanned for luminescence in a Tecan scanner.

### Polysome profile analysis and fractionation

Polysome profiling was performed as described previously (*20*). Briefly, 5 × 10^6^ to 8 × 10^6^ cells were washed with PBS containing CHX (100 μg/ml) and resuspended in 200 μl of hypotonic buffer [1.5 mM KCl, 2.5 mM MgCl_2_, and 5.0 mM tris-Cl (pH 7.4)] and 200 μl of hypotonic lysis buffer [same with 2% sodium deoxycholate, 2% Triton X-100, and 2.5 mM dithiothreitol (DTT)] plus CHX and gently disrupted using a Dounce homogenizer. The lysates were centrifuged at 8000*g* for 10 min at 4°C. The optical density (OD) of supernatant sample was measured on Ultrospec UV/Visible Spectrophotometer 1000 (Pharmacia Biotech). Equal OD of the supernatant was supplemented with 80 μl of heparin. Linear 10% to 50% sucrose gradients [80 mM NaCl, 5 mM MgCl_2_, 20 mM tris-Cl (pH 7.4), and 1 mM DTT] were prepared manually. The supernatant was loaded onto the pre-prepared sucrose gradients. Gradients were placed onto a pre-cooled SW41 Ti rotor (Beckman Coulter) and centrifuged at 39,000 rpm for 2.45 hours at 4°C using Optima XPN-80 Ultracentrifuge (Beckman Coulter). Following centrifugation, gradients were replaced onto a gradient density fractionation system (Brandel) and separated through a live OD 254 nm using a UA-6 UV/Vis detector (Teledyne Isco). Polysome fractions were collect using a Foxy R1 fraction collector (Teledyne Isco).

### RNA isolation and qRT-PCR

Total RNA was isolated from cells using the QIAGEN RNeasy Kit according to the manufacturer’s protocol. Polysomal RNA was isolated from polysome fractions by TRIzol (Invitrogen) according to the manufacturer’s protocol. Reverse transcription and real-time PCR (RT-PCR) from total RNA was carried out as described previously (*20*). The SYBR Green Dye detection system was used for quantitative real-time PCR on LightCycler 480 (Roche, Switzerland). Gene-specific primers (Microsynth AG, Switzerland) used are listed in table S6. Reactions containing ddH_2_O instead of cDNA were used as negative controls for target and housekeeping genes.

### tiRNA gel electrophoresis

HeLa and K562 cells were cultured in six-well plates. For positive control, cells were treated with 250 μM sodium arsenite for 2 hours. Total RNA was isolated from all samples as described above and heated with RNA loading dye at 60°C for 5 min. Then, RNA was separated on 15% urea polyacrylamide gel electrophoresis (PAGE) gel and tiRNAs detected as described previously (*57*).

### RNA-seq experiments and bioinformatic data analysis

RNA-seq from total RNA was reported previously (*20*). For polysome RNA sequencing, between 200 ng and 1 μg of high-quality RNA (RNA integrity number > 7) samples were used for cDNA synthesis and library preparation using the TruSeq Stranded Total RNA Sample Preparation Kit (Illumina). The cDNA libraries were then sequenced with 2 × 150–base pair reads on an Illumina HiSeq3000 instrument. The quality of the RNA-seq data was assessed using fastqc v.0.11.2 (Babraham Bioinformatics), RSeQC v.2.6.1 (*58*), and Qualimap v.2.2.1 (*59*). The readings were mapped to the human reference genome (assembly GRCh38) using HiSat2 v.2.1.0 (*60*). We then used Feature Counts from Subread v.1.5.3 (*61*) to count the number of readings overlapping with each gene as specified in the Ensembl annotation. For each total RNA and polysome RNA-seq data, the Bioconductor package DESeq2 v.1.18.0 (*62*) was used separately to test for the differential gene expression between RNH1-deficient cells and controls. TopGo was used to identify GO terms containing unusually many differentially expressed genes (*63*). From the above gene expression file, total genes (4029) with a cutoff of *P*_adj_ < 0.1 in polysomal RNA-seq and total-seq were selected. This includes the expression of ribosomal (152) and nonribosomal genes (3877) in WT and RNH1-deficient (KO) K562 cells. The values of log_2_(expression in total RNA-seq of WT) were plotted against the log_2_(expression in total RNA-seq of KO) using custom Python script. Similarly, the values of log_2_(expression in polysome RNA-seq of WT) were plotted against the log_2_(expression in polysome RNA-seq of KO). Dotted lines separate genes with log_2_(fold change) <1 and >1. Joint analysis of the polysomal and total RNA was performed with anota2seq v.1.0.1 in R v.3.4.2 to assign genes to different regulatory modes (*32*). ANOTA filtered genes (262) were plotted as described in the above paragraph.

To find the change in the translational activity of genes, 4029 genes with a cutoff of *P*_adj_ < 0.1 in polysomal RNA-seq and total RNA-seq were selected. The TA of these genes was calculated as polysomal RNA/Total RNA. The values of log_2_(TA) were plotted with log_2_(total RNA). Dotted lines separate genes with log_2_(fold change) <1 and >1. The translational activity of ANOTA filtered genes (262) was also calculated and plotted as described in the above paragraph.

### Immunoblot analysis

Cells from different cell lines, mouse total bone marrow cells, and hepatocytes were resuspended in the lysis buffer [20 mM tris (pH 7.4), 150 mM NaCl, 1% (v/v) NP-40, 10 mM EDTA]. Extracts were used for immunoblot after removing debris. Nitrocellulose membranes were stained with Ponceau S to ensure equal protein loading. The antibodies used for immunoblots are listed in Table 1.

**Table 1.** Antibodies: Details of the antibodies used in the study.

Antibody	Reactivity	Manufacturer	Reference
Anti-RNH1 (A-9)	Human	Santa Cruz Biotechnology	sc-365783
Anti-RNH1 (C-10)	Mouse	Santa Cruz Biotechnology	sc-271725
Anti-GAPDH (G-9)	Human, mouse	Santa Cruz Biotechnology	sc-365062
Anti–β-actin	Human, mouse	Abcam	ab8227
Anti-ANG	Human	Santa Cruz Biotechnology	sc-74528
Anti-mTOR (7C19)	Human	Cell Signaling	2983T
Anti–P-mTOR (Ser^2448^)	Human	Cell Signaling	5536T
Anti–P–4E-BP1 (Thr^37/46^)	Human	Cell Signaling	2855T
Anti–4–EB-P1	Human	Cell Signaling	9644T
Anti–p70 S6 kinase (Thr^389^)	Human	Cell Signaling	9234T
Anti–p70 S6 kinase	Human	Cell Signaling	sc-8418
Anti-RPL11	Human, mouse	Santa Cruz Biotechnology	sc-25931
Anti-RPS3	Human, mouse	Santa Cruz Biotechnology	sc-376008
Anti-RPS21	Human, mouse	Santa Cruz Biotechnology	sc-514411
Anti-H2A	Human	Cell Signaling	12349T
Anti-RPS19	Human, mouse	Santa Cruz Biotechnology	sc-100836
Anti-PABP1	Human	Santa Cruz Biotechnology	sc-32318
Anti-eIF4E	Human	Santa Cruz Biotechnology	sc-9976
Anti-eIF4G	Human	Cell Signaling	2469T
Anti-LARP1	Human	Santa Cruz Biotechnology	sc-515873
Anti-eIF2a	Human, mouse	Santa Cruz Biotechnology	sc-133132
Anti–P-eIF2a	Human, mouse	Cell Signaling	9721

### Co-IP experiments

WT or RNH1-KO cells were resuspended in the lysis buffer [20 mM tris (pH 7.4), 100 mM NaCl, 1% (v/v) Triton X-100, 10 mM EDTA]. Dynabeads were incubated with anti-PABP1 or anti-eIF4E antibody for 20 min and then washed with PBS + Tween 20. Subsequently, antibody-bound Dynabeads were incubated overnight with the cell lysate at 4°C. After washing, beads were resuspended in sample buffer and analyzed via immunoblot.

### Cap-binding assay

For cap-binding assay, cell lysates were prepared as described above. Then, 50 μl of the blank agarose bead control slurry and 50 μl of the γ-aminophenyl-m^7^GTP agarose C10-linked bead slurry (Jena Biosciences) were taken to separate 1.5-ml microcentrifuge tubes, centrifuged, and washed. Cell lysates were incubated with blank beads for 10 min at 4°C. Then, this mixture was centrifuged, and supernatant was transferred to the tube containing γ-aminophenyl-m^7^GTP agarose C10-linked beads. Beads and lysates were incubated together overnight at 4°C on a rotor. Then, the beads were washed, resuspended in sample buffer, and analyzed via immunoblot.

### MS analyses

WT or RNH1-KO human THP1, K562, and HEK293T cells (5 × 10^6^) were resuspended in the lysis buffer [20 mM tris (pH 7.4), 150 mM NaCl, 1% (v/v) NP-40, 10 mM EDTA]. Extracts were immunoprecipitated with anti-RNH1 magnetic beads and then were assessed by immunoblot. For MS analyses, beads after IP were washed with PBS and resuspended in 30 μl of 8 M urea and 50 mM tris-HCl (pH 8) and proteins were reduced (10 mM DTT, at 37°C for 30 min), followed by alkylation with 50 mM iodoacetamide (at 37°C for 30 min in the dark) and digestion by 100 ng of trypsin overnight at room temperature after dilution of urea to 1.3 M with 20 mM tris-HCl and 2 mM calcium dichloride (pH 8.0). The digests were acidified with trifluoroacetic acid and analyzed by nano–liquid chromatography (LC)–MS/MS (PROXEON coupled to a QExactive HF mass spectrometer, Thermo Fisher Scientific) with three injections of 5-μl digest each. Peptides were trapped on a micro-precolumn C18 PepMap100 (5 μm, 100 Å, 300 μm × 5 mm, Thermo Fisher Scientific, Reinach, Switzerland) and separated by backflush onto the analytical nano-column (C18, 3 μm, 155 Å, 0.075 mm inside diameter × 150 mm length, Nikkyo Technos, Tokyo, Japan) applying a 40-min gradient of 5% acetonitrile to 40% in water and 0.1% formic acid at a flow rate of 350 nl/min. The full-scan method was set with resolution at 60,000 with an automatic gain control (AGC) target of 1 × 10^6^ and maximum ion injection time of 50 ms. The data-dependent method for precursor ion fragmentation was applied with the following settings: resolution 15,000, AGC of 1 × 10^5^, maximum ion time of 110 ms, mass window 1.6 mass/charge ratio (*m*/*z*), collision energy 27, under fill ratio 1%, charge exclusion of unassigned and 1+ ions, peptide match preferred, and dynamic exclusion of fragmented precursors for 20 s, respectively.

MS data were interpreted with MaxQuant (version 1.5.4.1) (*64*) against a forward and reversed Swiss-Prot human protein sequence fasta file (release December 2017) using the following settings: strict trypsin cleavage rule allowing up to three missed cleavage sites; variable modifications of acetylated protein N termini and oxidation of methionine and fixed carbamidomethylation of cysteines; peptide and fragment mass tolerances of 10 and 20 parts per million (ppm); and match between runs of the same sample type activated, but not between different types, to avoid over-interpretation. Identified peptides and proteins were filtered to a 1% false discovery rate based on reversed database matches, and a minimum of two razor or unique peptides were requested for acceptance of protein group identification.

### Phylogenetic tree reconstruction

Human RNH1 DNA sequence (NM_203387.3) was used as a query to perform nucleotide Blast with selected species. From Blast output, only those pairs that had query cover more than 70% (except for nonmammalian species) and percent identity more than 60 were selected. No significant similarity was found below class “Reptilia” with above criteria. DNA sequences of selected species with these criteria were aligned using MAFFT multiple sequence aligner (*65*). Phylogenetic analysis was performed on IQ-Tree webserver (http://iqtree.cibiv.univie.ac.at/) using ultrafast bootstrap analysis with 1000 bootstrap alignments. Phylogenetic tree was visualized using Interactive Tree Of Life (iTOL). Sequences used for multiple sequence alignment are as follows: NM_203387.3 *Homo sapiens*; XM_024241348.1 *Pongo abelii*; XM_004050348.3 *Gorilla gorilla gorilla*; NM_001009060.1 *Pan troglodytes*; XM_001488475.4 *Equus caballus*; NM_145135.3 *Mus musculus*; NM_001047113.1 *Rattus norvegicus*; NM_001006473.1 *Gallus gallus*; XM_019485196.1 *Alligator mississippiensis*; XM_008120593.1 *Anolis carolinensis*.

### ITC experiments

The interaction analysis between RNH1 and PABPC1-A_24_, as well as between A_24_ and PABPC1, was performed using ITC (*66*) and MicroCal PEAQ-ITC (Malvern Instruments). Human PABPC1 was expressed and purified as previously reported (*41*). RNH1 and A_24_ were purchased from Promega (Madison, WI, USA) and Gene Design Inc. (Osaka, Japan), respectively. All samples were prepared in buffer containing 10 mM Na_2_HPO_4_ (pH 7.4), 1.8 mM KH_2_PO_4_, 137 mM NaCl, and 2.7 mM KCl. The experiments were performed at 25°C. PABPC1-A_24_ (8.9 μM) and PABPC1 (1.0 μM) were titrated with RNH1 (89 μM) and poly(A) (A_24_) (9.7 μM), respectively. A “One Set of Sites” model was applied for curve fitting. Thermodynamic parameters with error values were calculated from the fitted data.

### Statistical analysis

Data were expressed as means ± SEM of at least three biological replicates or three independent replicates from each genotype. Comparison between two groups was performed by two-tailed *t* test. A value of *P* < 0.05 was considered to be statistically significant. All statistical analyses were calculated using GraphPad Prism V. 9.5.1. No statistical methods were used to predetermine sample size. The experiments were not randomized, and the investigators were not blinded to allocation during experiments and outcome assessment. Polysome RNA-seq experiments were performed using three independent replicates from each genotype. MS studies were also performed on three independent replicates.
