# Supplementary material for: Ribonuclease inhibitor and angiogenin system regulates cell type–specific global translation
Source: Sci Adv. 2024 May 31;10(22):eadl0320. doi: 10.1126/sciadv.adl0320 (PMC11141627; doi:10.1126/sciadv.adl0320)
Supplement: Supplementary file 1 — Figs. S1 to S8 Legends for tables S1 to S6 [file sciadv.adl0320_sm.pdf]

Science Advances
AAAS

Supplementary Materials for

Ribonuclease inhibitor and angiogenin system regulates cell type–specific global translation

Martina Stillinovic *et al.*

Corresponding author: Ramanjaneyulu Allam, allam.ramanjaneyulu@unibe.ch

Sci. Adv. **10**, eadl0320 (2024)
DOI: 10.1126/sciadv.adl0320

The PDF file includes:

Figs. S1 to S8
Legends for tables S1 to S6

Other Supplementary Material for this manuscript includes the following:

Tables S1 to S6

Fig. S1

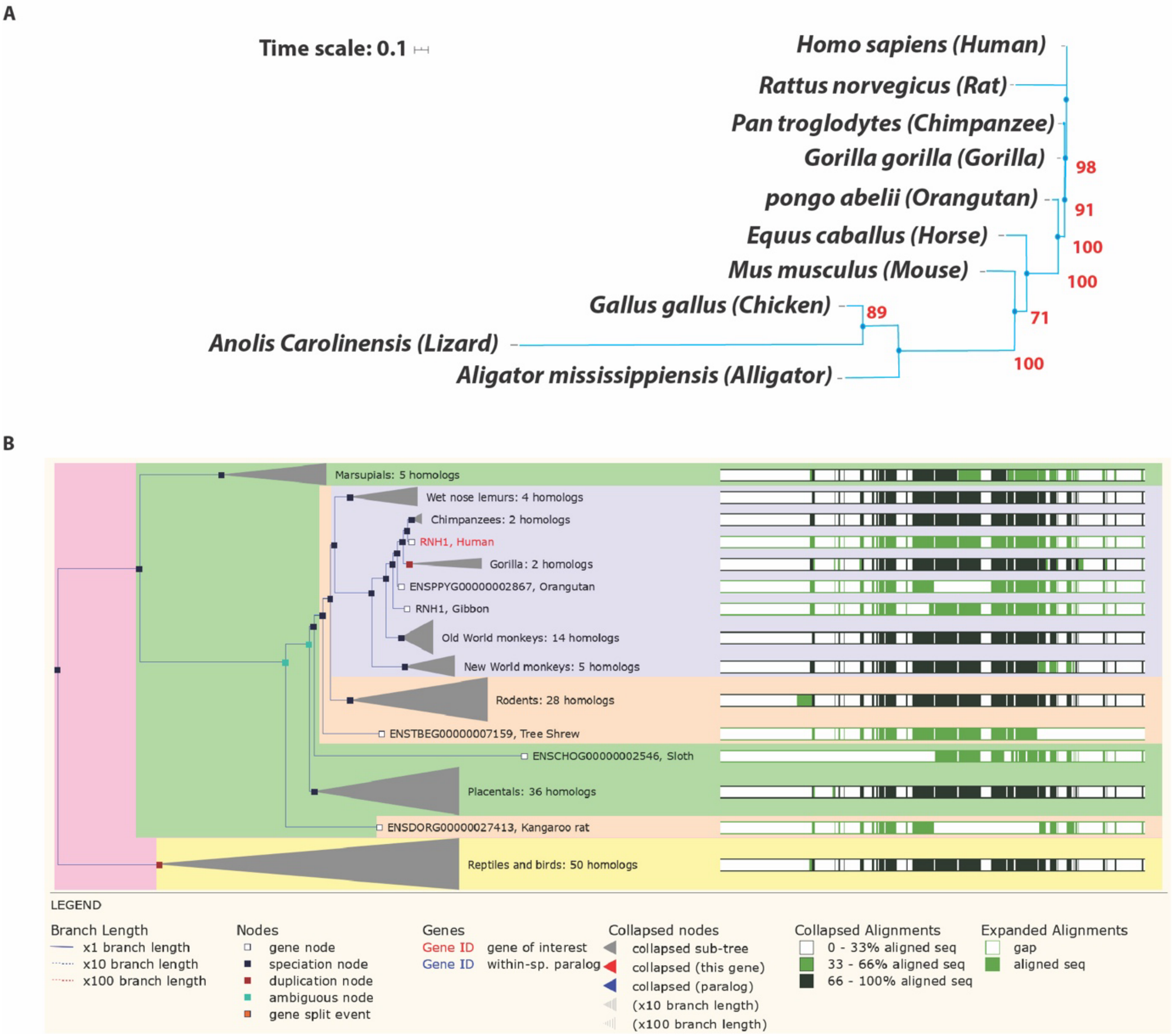

Fig S1. ***RNH1*** **gene evolved only in vertebrates**

(A) Phylogenetic tree representing the evolutionary relationships between vertebrate *RNH1* suggesting that it has diverged minimally among different subclasses of mammals. *RNH1* DNA sequence alignments were made using MAFFT. A maximum likelihood phylogenetic tree was generated using IQ-Tree with 1000 bootstrap replicates. Bootstrap values >70% are shown in red. **(B)** Ensembl gene tree for *RNH1* generated by the Gene Orthology/Paralogy prediction method. Gene trees are constructed using one representative protein for every gene in every species in Ensembl. The figure shows the maximum likelihood phylogenetic tree representing the evolutionary history of *RNH1* gene.

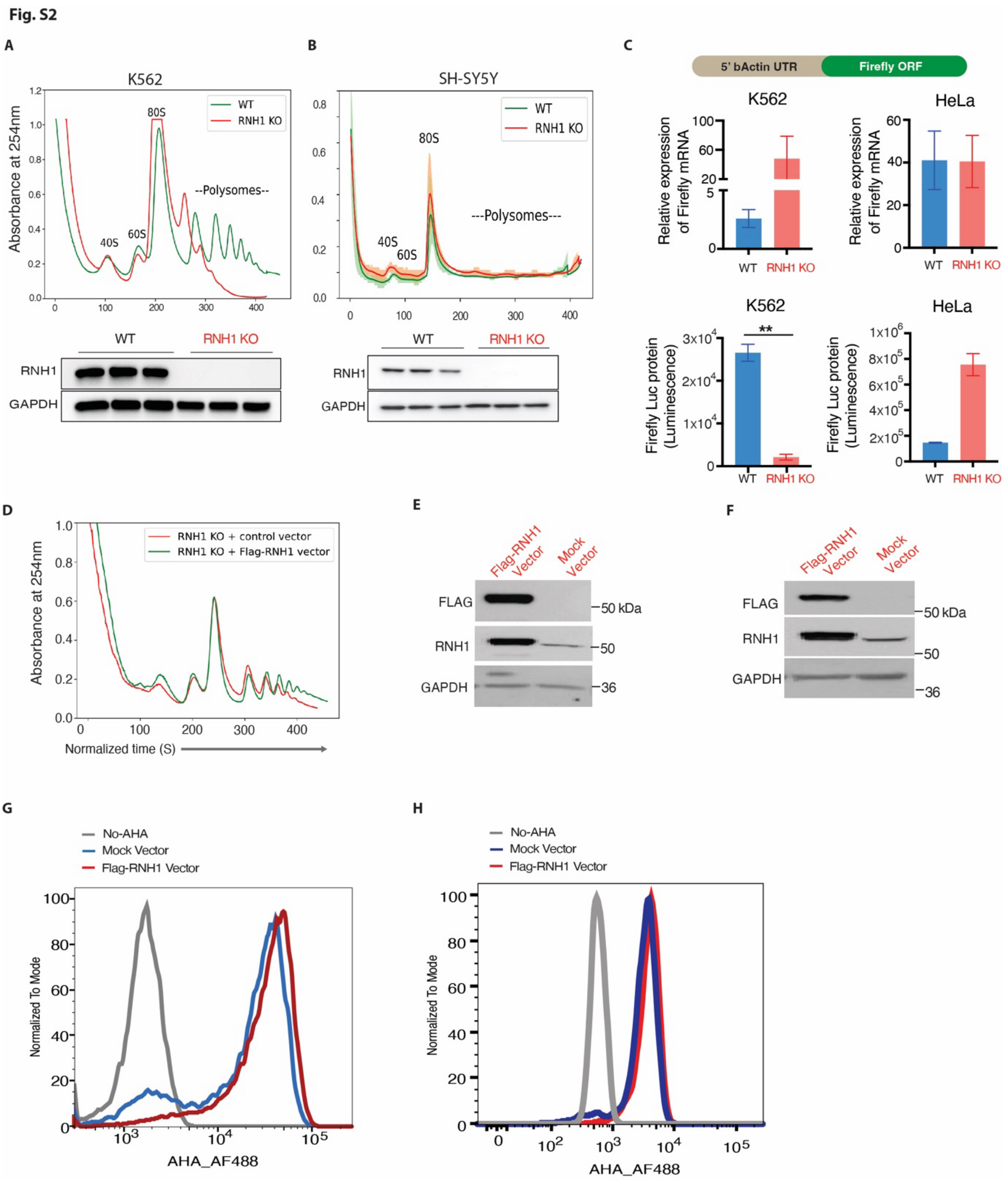

Figure S2. Loss of RNH1 decreases translation in hematopoietic cells but not in non-hematopoietic cells

(**A and B**) Sucrose gradient polysome profiles for WT and corresponding RNH1 KO of K562 or SH-SY5Y cells (N=3). Arrow shows the direction of the sucrose gradient from low to high density. Mean value of absorbance from three independent experiments plotted with the

standard deviation (upper panel). Total protein lysates of WT and RNH1 KO cells were analyzed by western blot with the indicated antibodies. Blots are representative of three independent experiments (lower panel). (**C**) Schematics of luciferase expressing plasmid with beta actin (*ACTB*) 5`UTR (upper panel). WT or RNH1 KO of HeLa or K562 cells were transfected with luciferase expressing plasmid. Cells were analysed for firefly mRNAs by qRT-PCR, normalized to 18S rRNA expression and luciferase protein expression by luciferase assay. Data are shown as mean ± SEM and are representative of 3 independent experiments. **p<0.01. (**D)** Sucrose gradient polysome profiles for control and FLAG-RNH1 expressing RNH1 KO K562 cells. Data is representative of 3 independent experiments. **(E and F**) Flag-RNH1 is over expressed in K562(E) and HeLa (F) cells. Cell lysates were analyzed by western blot with the indicated antibodies. Blots are representative of three independent experiments. (**G**) K562 cells were incubated for 1h with L-Azidohomoalanine (AHA) and FACS analysis were performed to measure AHA incorporation. Representative histograms were shown for AHA fluorescence. Data is representative of 3 independent experiments. (**H**) HeLa cells were incubated for 1h with L-Azidohomoalanine (AHA) and FACS analysis were performed to measure AHA incorporation. Representative histograms were shown for AHA fluorescence. Data is representative of 3 independent experiments.

Fig. S3

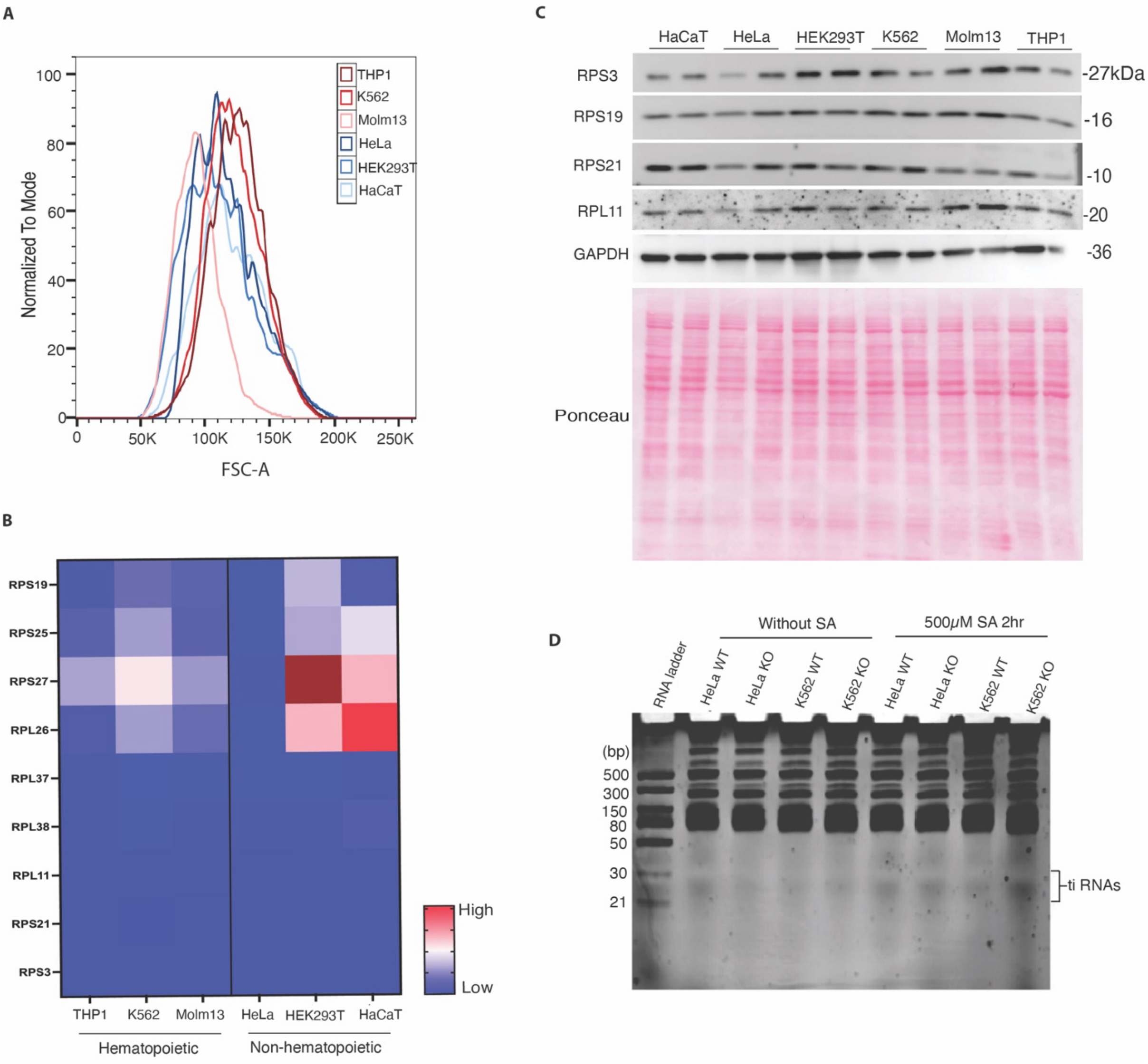

Figure S3. RNH1 mediated translation specificity is not dependent on cell size and RPs gene expression

(**A**) Flow cytometry analysis for forward scatter (FSC) to evaluate cell size from different hematopoietic and non-hematopoietic cells. (**B**) Heatmap showing qRT-PCR results of mRNA relative expression of different ribosomal protein genes from hematopoietic and non-hematopoietic cell lines. mRNA levels were normalized to beta-Actin mRNA. Results are representative of two independent experiments. (**C**) Total protein lysates of different hematopoietic and non-hematopoietic cell lines were analyzed by western blot with the

indicated antibodies. Blots are representative of three independent experiments. (**D**) HeLa and K562 cells of WT or RNH1 KO conditions were collected after 2hr with or without stimulation by 500μMsodium arsenite (SA) and RNA was isolated. 3μg of RNA was mixed with RNA loading dye and heated for 5min, 60°C and loaded on Urea-PAGE for tiRNA separation. Gels are representative of 3 independent experiments.

Fig. S4

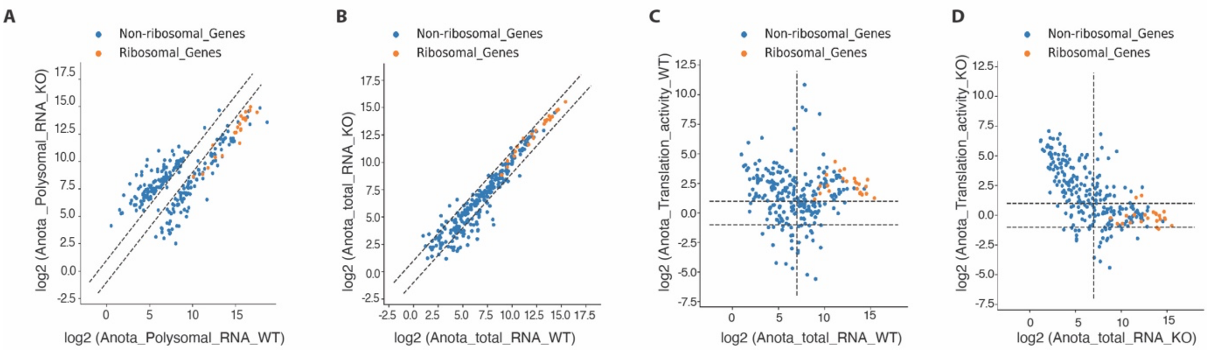

Figure S4. RNH1 mediates RPs gene translation

(**A and B**) Polysomal RNA-seq and total RNA-seq data of WT and RNH1 KO K562 cells were subjected to ANOTA analysis. The expression of ANOTA filtered ribosomal and non-ribosomal genes (Total 262 genes) with cutoff of padj. <0.1 in polysomal RNA-seq and total RNA-seq were selected and plotted. Dotted lines separate genes with log2(fold change) <1 and >1. (**C and D**) Plot of Log2 fold change of total RNA verses translational activity (TA) of ribosomal and non-ribosomal genes in WT (C) or RNH1 KO (D) samples. Horizontal dotted lines separate genes with log2(fold change) <1 and >1. Vertical line separate genes with arbitrary cutoff of >7< for log2 (expression in total RNA-seq).

Fig.S5

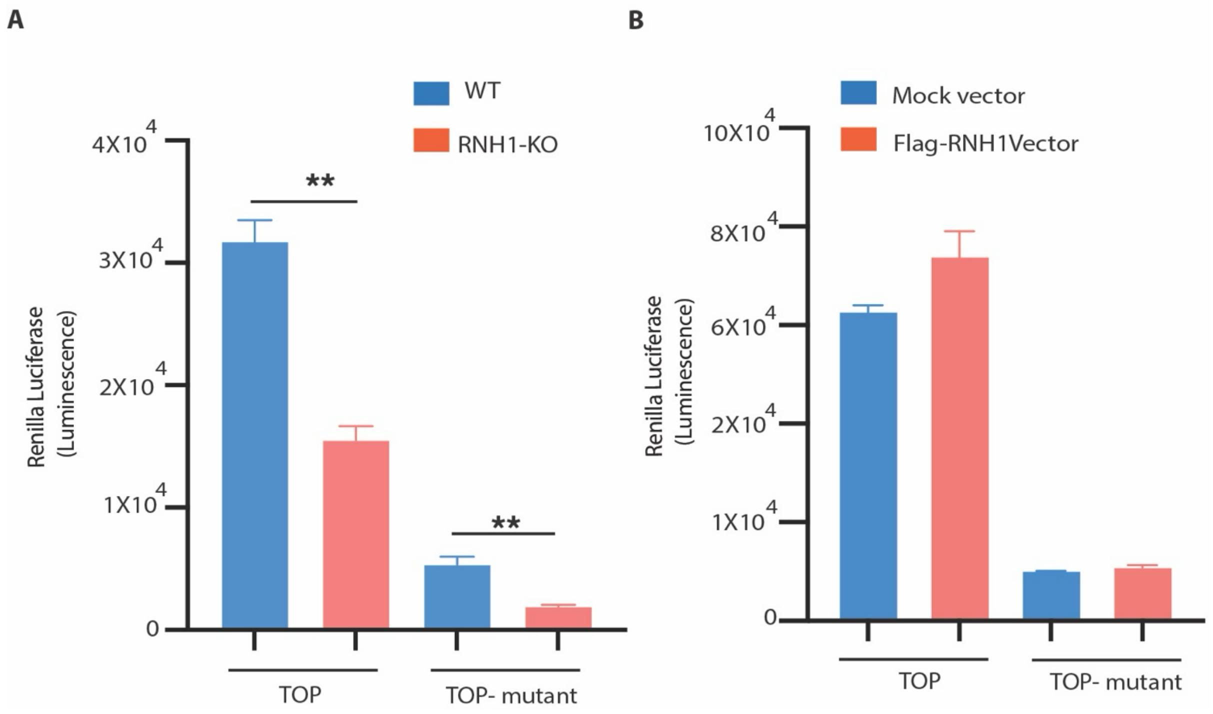

Figure S5. RNH1 mediates translation independent of TOP sequence

(**A and B**) WT and RNH1-KO (A) or mock and Flag-RNH1 overexpressing K562 cells (B) were transfected with Renilla reporter constructs containing the promoter and 5' UTRs of eEF2 with or mutant TOP sequence. After 24h, Cell lysates were then analyzed for Renella luciferase activity. Data are shown as mean ± SEM. Significance was determined by two tailed t-test. $^{**}p<0.01$.

Fig. S6

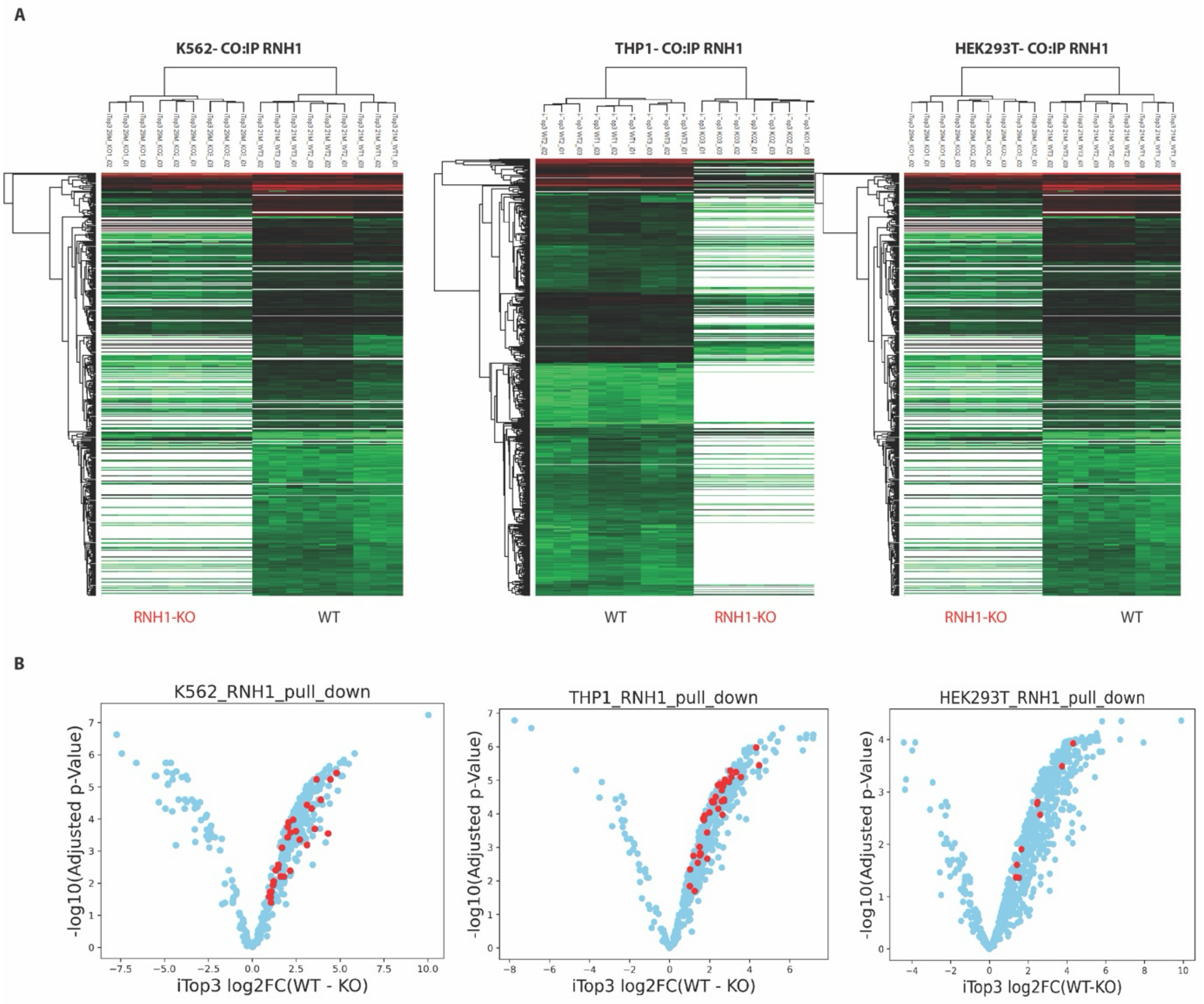

Figure S6. RNH1 binds to Ribosomal proteins

(**A**) Heatmap showing Cluster analysis with iTop3 values of RNH1 binding total proteome from WT THP1 or K562 or HEK293T cells compared with RNH1 KO corresponding cells. (probability of false positive < 0.1). (**B**) Volcano plot showing ribosomal proteins in red.

Fig. S7

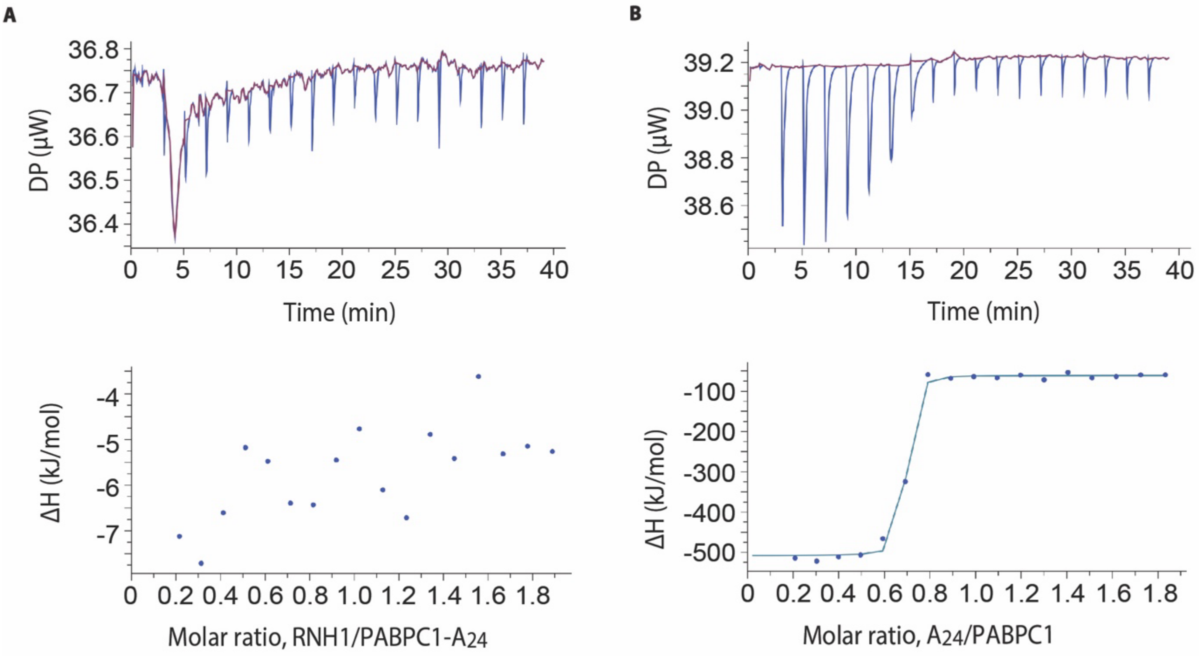

Figure S7. No interaction between PABPC1-A_{24} and RNH1 by ITC.

ITC analysis of the interactions of (**A**) PABPC1-A_{24} with RNH1, (**B**) PABPC1 with A_{24}. Upper panel, traces of the 19 titrations of 2 µL aliquots of 89 µM RNH1 into cells containing 8.9 µM PABPC1-A_{24} (A), and 9.7 µM A_{24} into cells containing 1 µM PABPC1 (B). Lower panel, the integrated binding isotherms obtained from the experiments were fitted using a "One Set of Sites" model. In the case of the PABPC1-A_{24} interaction (B), the following parameters were obtained from the best fit (solid line) with the error values calculated from the fitting; stoichiometry (N) = 0.649 ± 0.004, K_d = 0.4 ± 0.3 nM, ΔH = -447 ± 7 kJ/mol, ΔG = -53.9 kJ/mol, ΔS = -1.32 kJ/(mol·K).

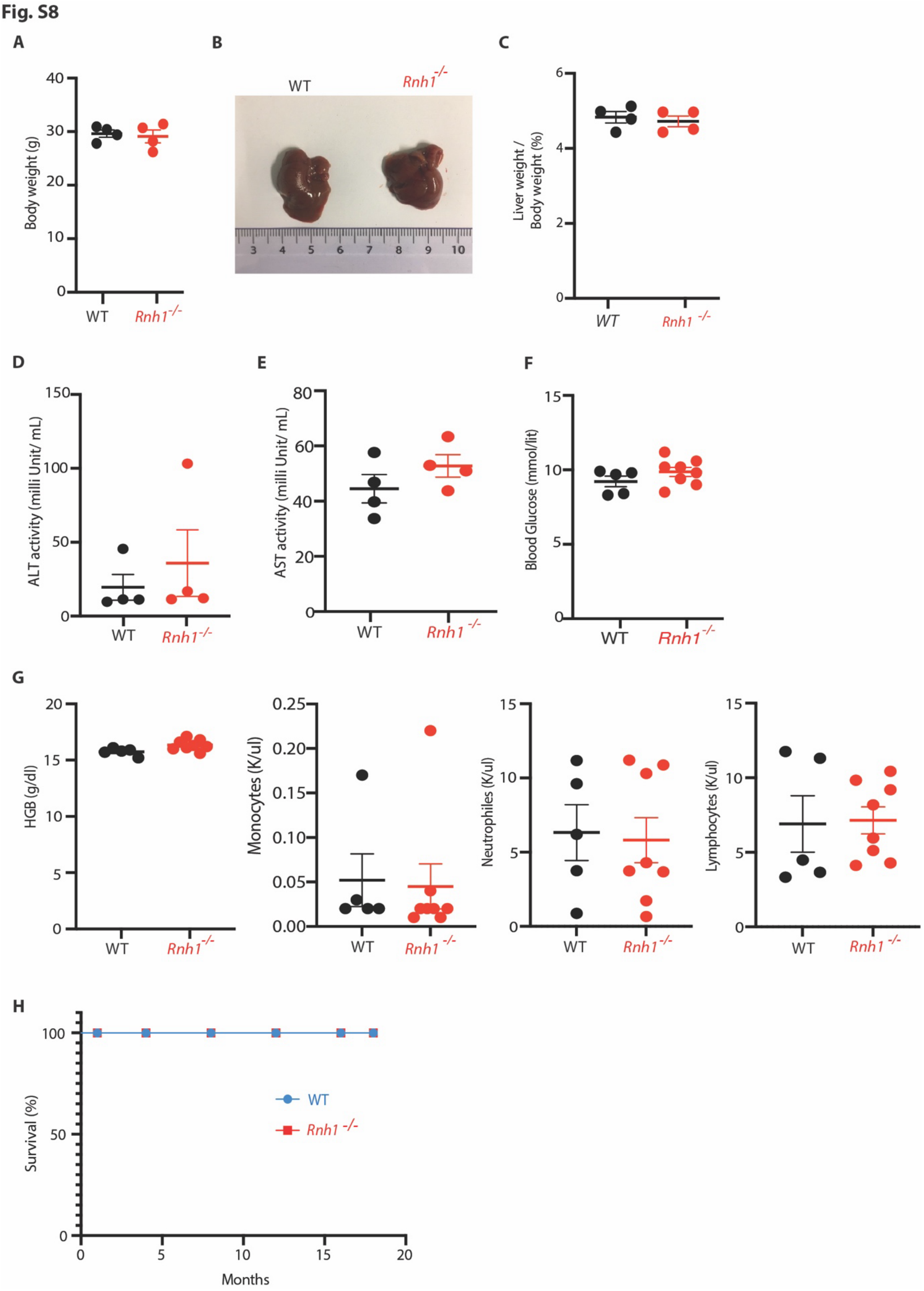

Fig S8. Loss of RNH1 in the liver does not affect liver function and overall survival

(A) Body weight of WT (*Rnh1* $^{fl/fl}$) and *Rnh1*$^{-/-}$ (*Rnh1* $^{fl/fl}$*, Alb-Cre*$^{+}$) mice at 14 weeks of age (N=4). **(B and C)** Representative image of liver and liver weight of WT and *Rnh1*$^{-/-}$ mice at 14 weeks (n = 4 mice). (**D-F**) Serum ALT, AST and glucose levels of WT and *Rnh1*$^{-/-}$ mice at 14 weeks (n = 4 mice). **(G)** Peripheral blood (PB) counts of neutrophils, granulocytes, lymphocytes, and hemoglobin (HBG) concentration in WT and *Rnh1*$^{-/-}$ mice at 14 weeks (n = 4 mice). All the data are shown as mean ± SEM. (**H**) Kaplan–Meier survival curves of WT and *Rnh1*$^{-/-}$ mice (n = 5 mice).

Table S1. (separate file)

Differential expression polysome vs Total RNA

Table S2. (separate file)

ANOTA Analysis polysome vs Total RNA

Table S3. (separate file)

Mass spec data from K562 cells

Table S4. (separate file)

Mass spec data from HEK293T

Table S5. (separate file)

Mass spec data from THP1 cells

Table S6. (separate file)

qPCR primer list
